# Engineering *Novosphingobium aromaticivorans* to produce *cis,cis*-muconic acid from biomass aromatics

**DOI:** 10.1128/aem.01660-23

**Published:** 2023-12-20

**Authors:** Avery C. Vilbert, Wayne S. Kontur, Derek Gille, Daniel R. Noguera, Timothy J. Donohue

**Affiliations:** 1 Great Lakes Bioenergy Research Center, University of Wisconsin-Madison, Madison, Wisconsin, USA; 2 Wisconsin Energy Institute, University of Wisconsin-Madison, Madison, Wisconsin, USA; 3 Department of Civil and Environmental Engineering, University of Wisconsin-Madison, Madison, Wisconsin, USA; 4 Department of Bacteriology, University of Wisconsin-Madison, Madison, Wisconsin, USA; Michigan State University, East Lansing, Michigan, USA

**Keywords:** aromatic compounds, carbon metabolism, sphingomonads, muconic acid, *Novosphingobium*, metabolic engineering, extradiol, intradiol, lignin, decarboxylases

## Abstract

The platform chemical *cis,cis-*muconic acid (*cc*MA) provides facile access to a number of monomers used in the synthesis of commercial plastics. It is also a metabolic intermediate in the β-ketoadipic acid pathway of many bacteria and, therefore, a current target for microbial production from abundant renewable resources via metabolic engineering. This study investigates *Novosphingobium aromaticivorans* DSM12444 as a chassis for the production of *cc*MA from biomass aromatics. The *N. aromaticivorans* genome predicts that it encodes a previously uncharacterized protocatechuic acid (PCA) decarboxylase and a catechol 1,2-dioxygenase, which would be necessary for the conversion of aromatic metabolic intermediates to *cc*MA. This study confirmed the activity of these two enzymes *in vitro* and compared their activity to ones that have been previously characterized and used in *cc*MA production. From these results, we generated one strain that is completely derived from native genes and a second that contains genes previously used in microbial engineering synthesis of this compound. Both of these strains exhibited stoichiometric production of *cc*MA from PCA and produced greater than 100% yield of *cc*MA from the aromatic monomers that were identified in liquor derived from alkaline pretreated biomass. Our results show that a strain completely derived from native genes and one containing homologs from other hosts are both capable of stoichiometric production of *cc*MA from biomass aromatics. Overall, this work combines previously unknown aspects of aromatic metabolism in *N. aromaticivorans* and the genetic tractability of this organism to generate strains that produce *cc*MA from deconstructed biomass.

**IMPORTANCE:**

The production of commodity chemicals from renewable resources is an important goal toward increasing the environmental and economic sustainability of industrial processes. The aromatics in plant biomass are an underutilized and abundant renewable resource for the production of valuable chemicals. However, due to the chemical composition of plant biomass, many deconstruction methods generate a heterogeneous mixture of aromatics, thus making it difficult to extract valuable chemicals using current methods. Therefore, recent efforts have focused on harnessing the pathways of microorganisms to convert a diverse set of aromatics into a single product. *Novosphingobium aromaticivorans* DSM12444 has the native ability to metabolize a wide range of aromatics and, thus, is a potential chassis for conversion of these abundant compounds to commodity chemicals. This study reports on new features of *N. aromaticivorans* that can be used to produce the commodity chemical *cis,cis*-muconic acid from renewable and abundant biomass aromatics.

## INTRODUCTION

One strategy to increase the environmental and economic sustainability of chemical production relies on harnessing the native or engineered metabolic pathways of microbes to catalyze the conversion of renewable resources into valuable products. Advances in genomics have enabled metabolic engineering approaches to convert abundant renewable resources into a number of targets for bioproduct production. One of these bio-privileged molecules is *cis,cis*-muconic acid (*cc*MA), which can be used as a precursor for the production of polymers including nylon-6,6, polyurethane, and polyethylene terephthalate (1). This dicarboxylic acid is an intermediate in the β- ketoadipic acid pathway of many bacteria and thus its production is amenable to metabolic engineering approaches.

The biological production of *cc*MA has been reported from food-grade, non-renewable sugars like glucose (2). Recently, significant attention has been drawn toward the production of *cc*MA from biomass lignin (3–6). Lignin is the most abundant renewable source of aromatics on the planet (7, 8) and accounts for approximately 20%–30% (wt/wt) of dry biomass (9–11). However, lignin remains an underutilized industrial carbon source due to the chemical heterogeneity of the lignocellulose polymers. Lignin is composed of phenolic monomers that contain either two methoxy groups (S), one methoxy group (G), or no methoxy group (H) on the aromatic ring (12). Furthermore, biomass deconstruction methods used to recover aromatics produce a diverse set of aromatic monomers, dimers, and oligomers (13). Therefore, metabolic engineering techniques for conversion of diverse biomass aromatics into simple product streams can be attractive if the hosts can funnel S, H, and G aromatics through common intermediates, thereby alleviating some of the challenges associated with plant cell wall heterogeneity (14–16).

**Fig 1:**
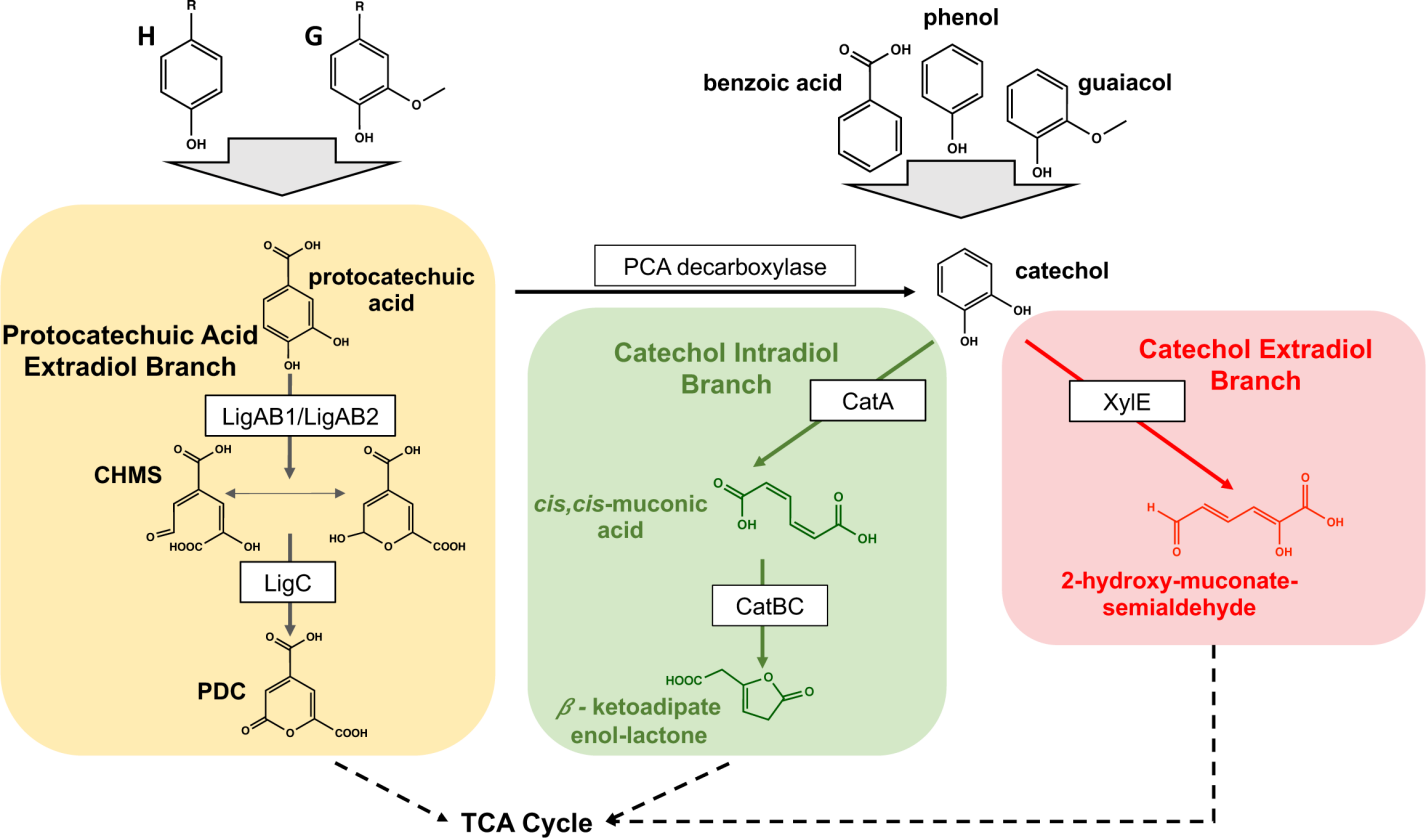
Metabolic pathways for aromatic catabolism from H and G phenolic monomers and other aromatics (benzoic acid, phenol, and guaiacol, right). Two central intermediates, PCA and catechol of their metabolism, are shown.

In order to achieve this goal, host selection for metabolic funneling techniques remains an important factor in the conversion of heterogeneous biomass aromatics to commodity chemicals. Strategies for biological *cc*MA production from aromatics typically relies on the intradiol cleavage of catechol by a catechol 1,2-dioxygenase, CatA. Catechol is a known intermediate in the aromatic metabolism of benzoic acid, guaiacol, and phenol (Fig. 1) (17, 18). Pathways for production of *cc*MA from biomass-derived H- and G-type aromatics have often been designed to divert the pathway intermediate protocatechuic acid (PCA) to catechol via heterologous expression of a PCA decarboxylase from either *Klebsiella pneumoniae* or *Enterobacter cloacae* (5, 6, 19). Thus far, *Pseudomonas putida* KT2440 is a well-described bacterial host strain for *cc*MA production from either pure aromatics (*p*-coumaric or ferulic acids) or deconstructed biomass (20). Engineering of *P. putida* KT2440 for efficient *cc*MA production required the use of exogenous promoters for expression of foreign genes and deletion of a gene that encodes a global catabolic transcriptional regulator (20, 21) to overcome metabolic bottlenecks that included accumulation of several pathway intermediates [PCA, vanillin, and 4*-*hydroxybenzoic acid (4-HBA)] (6, 22, 23). Therefore, we sought to assess the ability of other bacterial hosts for producing *cc*MA from biomass aromatics by leveraging metabolic and genetic features of the aromatic metabolizing bacterium *Novosphingobium aromaticivorans* DSM12444.

*N. aromaticivorans* is one of several sphingomonads that are being studied as a potential chassis for production of chemicals from biomass aromatics (4, 24). This α-protobacterium, isolated from a polyaromatic hydrocarbon-contaminated site, can utilize many aromatics as a sole carbon source (25–27). *N. aromaticivorans* and other sphingomonads have the native ability to cleave major inter-subunit linkages of lignin aromatic oligomers allowing these microbes to convert many mixed aromatics into products (11, 28–31). The genetic tractability of *N. aromaticivorans* has enabled the engineering of mutant strains that can stoichiometrically produce the commodity chemical 2-pyrone-4,6-dicarboxylic acid (PDC) from native G, H, and S aromatics or aromatic diketones that are generated during lignin deconstruction (30–32). These characteristics of *N. aromaticivorans* make it attractive for microbial funneling of the heterogeneous mixture of aromatics in deconstructed biomass. However, knowledge gaps still remain on the number and diversity of enzymes that compose this bacterium’s aromatic metabolic pathways. This work sought to address some of these knowledge gaps by investigating the aromatic metabolism of *N. aromaticivorans* and assessing its feasibility to serve as a host for *cc*MA production from biomass aromatics.

Here, we evaluated the potential of engineering *N. aromaticivorans* as a chassis for *cc*MA production from lignin biomass. The genome of *N. aromaticivorans* predicts that it encodes a previously uncharacterized PCA decarboxylase and a CatA enzyme which could be used to divert PCA to catechol for subsequent production of *cc*MA from catechol (Fig. 1). We used *in vitro* assays to confirm the activity of these putative enzymes and applied this information to generate defined mutants that test the predicted pathway for conversion of PCA to *cc*MA by *N. aromaticivorans*. Through the combination of these *in vitro* assays and the analysis of defined mutants, we identified a previously unreported PCA catabolic pathway in *N. aromaticivorans*. This information differentiates *N. aromaticivorans* from other reported *cc*MA production hosts which lack a native decarboxylase for conversion of PCA to catechol (20). We also asked whether the expression of these native genes could compete with expression of heterologous genes used previously for *cc*MA production by placing individual genes under control of native aromatic-inducible promoters in the genome. Overall, this study revealed the ability to engineer *N. aromaticivorans* to stoichiometrically convert PCA to *cc*MA in batch cultures and to produce greater than 100% yields of *cc*MA from the measured aromatics in biomass alkaline pretreatment liquor (APL). It also highlights that an engineered *N. aromaticivorans* strain derived solely from native genes produces *cc*MA at a similar rate and production yield as seen with a *N. aromaticivorans* strain that heterologously expresses the enzymes from *E. cloacae* commonly used in other work (4–6, 33). This study expands our understanding of the aromatic metabolism of *N. aromaticivorans* and demonstrates the feasibility of this host for production of *cc*MA from biomass-derived aromatics.

## RESULTS

### PCA catabolism in *N. aromaticivorans*

The genome sequence of *N. aromaticivorans* predicts that it encodes enzymes which can convert both H and G biomass aromatics into PCA (25, 30, 31). Therefore, in order to test if we could engineer a strain that produces *cc*MA from H and G lignin aromatics, we first sought to develop further understanding of PCA catabolism in *N. aromaticivorans*. It was previously shown that the PCA 4,5 extradiol cleavage pathway is the major pathway in *N. aromaticivorans* when these cultures are supplied only an aromatic substrate (31). Additionally, it was shown that there are two 4,5 PCA dioxygenase homologs (LigAB1 and LigAB2) of the PCA extradiol cleavage pathway that can convert PCA to 4-carboxy-2-hydroxy-*cis,cis*-muconate-6-semialdehyde (Fig. 1) (31). Deletions of these genes predicted to encode enzymes in the PCA 4,5 extradiol cleavage pathway (Fig. 1) resulted in a *N. aromaticivorans* strain that accumulates extracellular PDC from H and G aromatics with yields greater than 70% when supplied an aromatic and glucose. These results agree with published data and demonstrate that this is a major pathway for the catabolism of H and G aromatics (30, 31). However, previous work also found less than stoichiometric yields of PDC when cells were grown with PCA as a carbon source (30). These results suggest that LigAB1 and LigAB2 play a significant role in PCA metabolism, but that the existence of another PCA-consuming pathway could be the cause of the decreased yield of PDC when this strain is grown with PCA. Based on these results, we hypothesized that blocking the *N. aromaticivorans* PCA extradiol cleavage pathway in the parent strain (12444, Table 1) would result in a strain that metabolizes PCA but at a rate slower than observed in cells that contain LigAB1 and LigAB2.

**TABLE 1:** *N. aromaticivorans* mutant strains used in this study

Strain name	Genotype	Source
12444	12444Δ1879	(30)
12444_ΔligAB1/2	12444Δ1879Δ*ligAB1*Δ*ligAB2*	(31)
12444_PCA	ΔligAB1/2 Δ*Saro3873-3878*	This study
LigAB1_EcDec	12444_PCA ΔligAB1:*ecdB/ecaroY/ecdD*	This study
LigAB1_NaDec	12444_PCA ΔligAB1:*nadBCD*	This study
EcDec_cat	LigAB1_EcDec Δ*xylE* Δ*catBC*	This study
NaDec_cat	LigAB1_NaDec Δ*xylE* Δ*catBC*	This study
EcDec_*cc*MA	EcDec_cat ΔxylE: *eccatA*	This study
NaDec_*cc*MA	EcDec_cat ΔxylE: *nacatA*	This study

**Fig 2:**
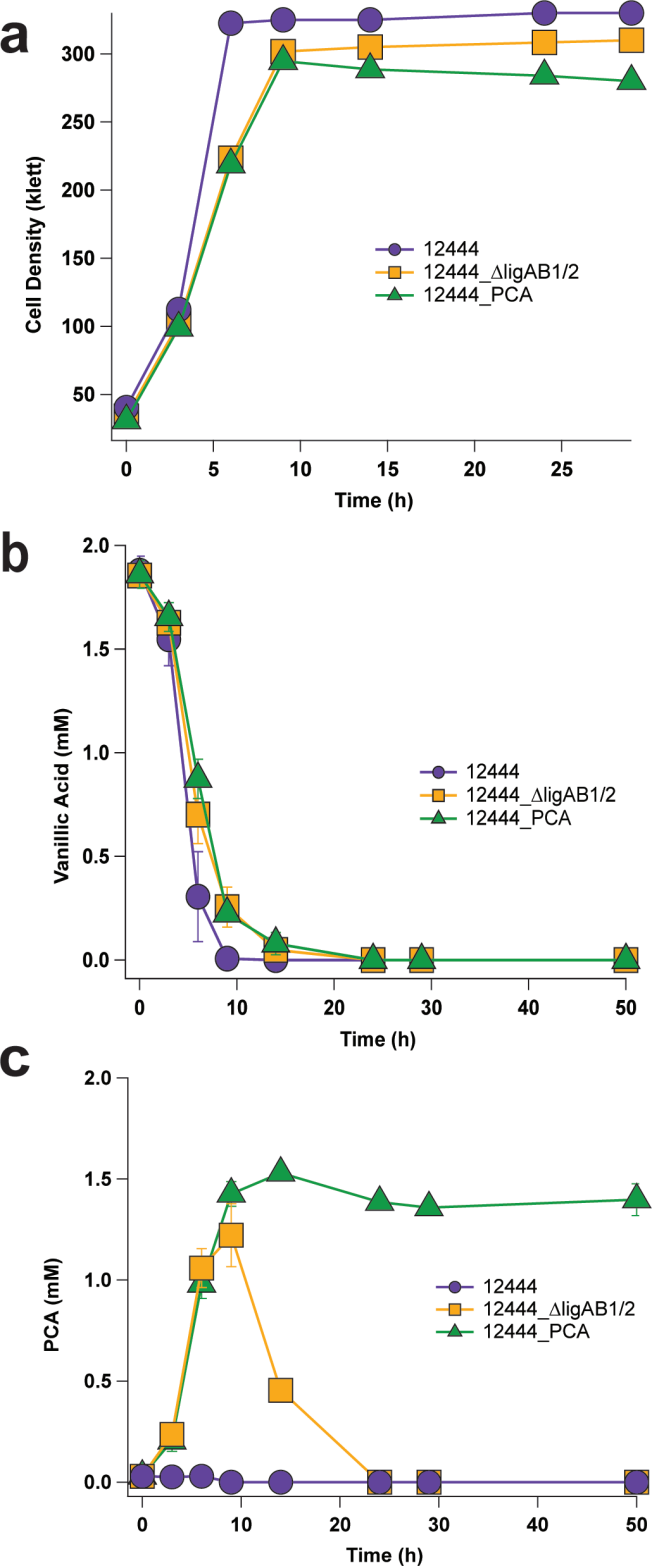
Cell density (a) and extracellular concentrations of vanillic acid (b) and PCA (c) in cultures of the parent strain 12444 (purple), the 12444_ΔligAB1/2 (yellow) strain, and the ΔligAB1/2 ΔnadBCD (12444_PCA, green) strain. Cells were grown in batch cultures in minimal media containing 2 mM vanillic acid and 10 mM glucose. No other aromatics were detected in the media. All experiments were performed in triplicate. Error bars represent one standard deviation above and below the mean.

To test this hypothesis, we determined the effect of deletions in *ligAB1* and *ligAB2* on the ability to metabolize PCA in the presence of glucose as an auxiliary carbon source. This strain 12444_Δ*ligAB*Δ*ligAB*2 (31) (hereafter called 12444_Δ*ligAB1/*2) and the parent strain 12444 (Table 1) were grown in media containing 2 mM vanillic acid, a G aromatic, and 10 mM glucose. Both strains grew to similar cell densities and fully eliminated the vanillic acid from the medium. As predicted, there was no extracellular accumulation of PCA in the 12444 parent strain (Fig. 2c; Fig. S5). However, the 12444_Δ*ligAB1/*2 mutant transiently accumulated detectable levels of PCA in the media (Fig. 2c; Fig. S5). The observation of the transient formation of PCA when the 12444_Δ*ligAB*1/2 strain is grown with vanillic acid is consistent with the hypothesis that another route for metabolism of this predicted pathway intermediate is present (30, 31).

To test how PCA might be metabolized in the 12444_Δ*ligAB*1/2 strain, we analyzed the genome of *N. aromaticivorans* for genes that encode homologs of enzymes known to catalyze PCA ring-opening reactions in other organisms. This analysis failed to identify genes that encode proteins with >25% amino acid sequence identity to the *Pseudomonas putida* 3,4 dioxygenase (PcaHG) which catalyzes intradiol cleavage of PCA to form 3-carboxy-*cis,cis*-muconate or to a 2,3 PCA dioxygenase (PraA) that produces 5-carboxy-2-hydroxymuconate-6-semialdehyde (34–36). However, this analysis showed that *N. aromaticivorans* contained genes (Saro*_*3873, Saro*_*3877, Saro_3878; hereafter referred to as *nadB*, *nadC*, and *nadD*, respectively) that encoded proteins with significant amino acid identity to the known B, C, and D gene products involved in PCA decarboxylation by *Klebsiella pneumoniae*, *Enterobacter cloacae,* and other bacteria (Table S3) (5, 6, 37–39). Based on these amino acid sequence identities and the fact that the transcripts encoding these genes are more abundant when cells are grown in the presence of aromatics than in the presence of glucose (32), we propose that a previously uncharacterized *N. aromaticivorans* PCA decarboxylase was responsible for metabolizing the PCA that transiently accumulated in the media of the 12444_Δ*ligAB1/2* strain. We performed a set of *in vitro* and *in vivo* experiments to test this proposal.

### Characterization of *N. aromaticivorans* PCA decarboxylase enzyme

The amino acid sequence of the predicted *N. aromaticivorans* PCA decarboxylase enzyme (hereafter called NadBCD) predicts that it is most similar to a family of hydroxyarylic acid decarboxylases (37) that typically require three proteins, BCD, for activity. The *B* gene product encodes a predicted prenyltransferase that produces a prenylated flavin mononucleotide (prFMN) cofactor, while *C* catalyzes decarboxylation and *D* encodes a protein of unknown function (40, 41). Analysis of the most extensively studied decarboxylases in this family (*Escherichia coli* UbiD and *Enterobacter cloacae* EcAroY) has shown that the prFMN cofactor is required for decarboxylase activity (38, 42). Therefore, we tested if the *N. aromaticivorans* NadCD had PCA decarboxylase activity and whether it required a prFMN cofactor for catalysis (38).

**Fig 3:**
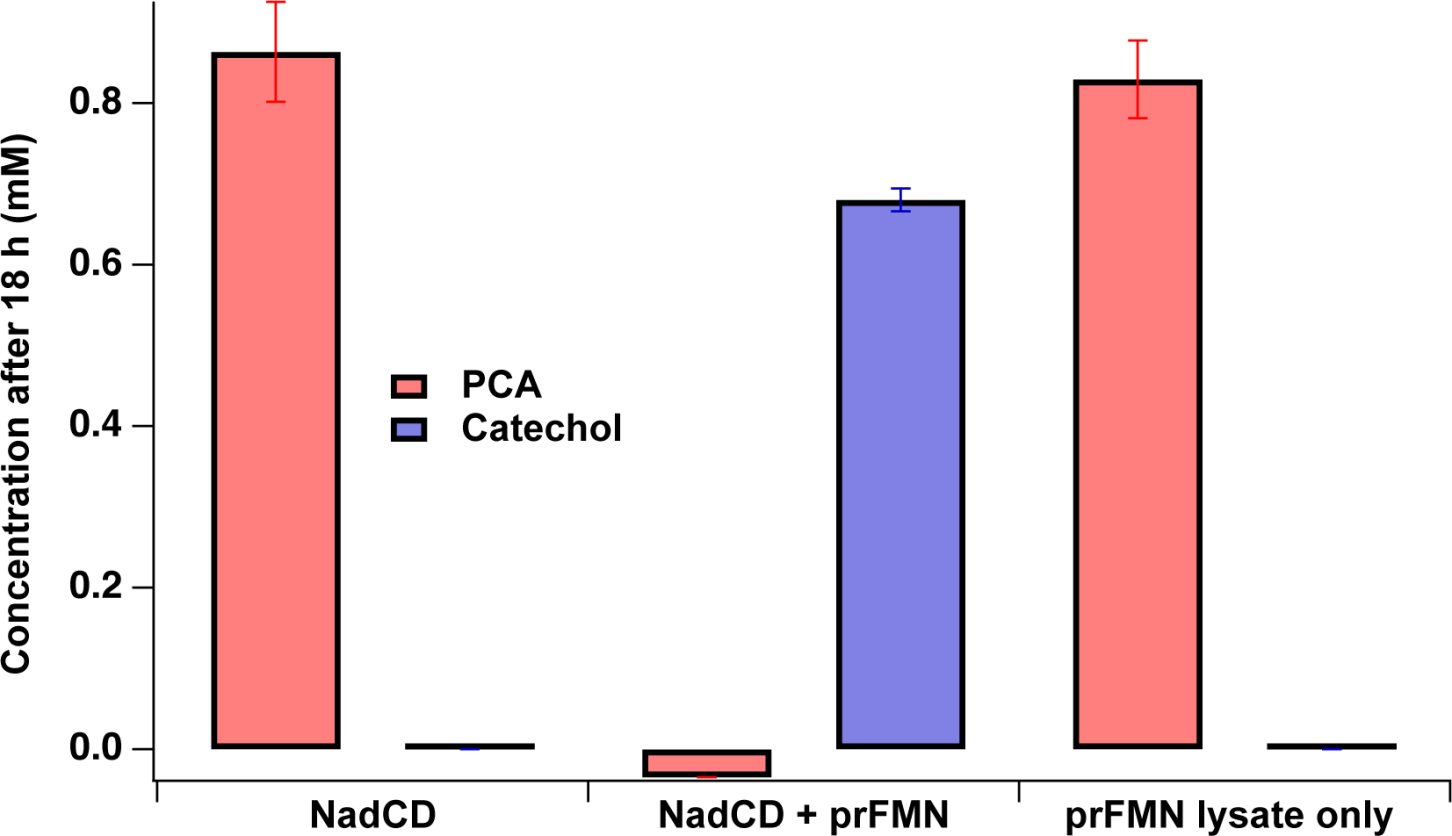
The dependence of NadCD on a prFMN source for PCA decarboxylase activity. Experiments were performed with 100 nM NadCD with 1 mM PCA in either the presence or absence of a prFMN lysate. The bars represent the concentration of either PCA (red) or catechol (blue) after 18 h of incubation at room temperature. A control of lysate with 1 mM PCA was also performed to ensure no PCA to catechol conversion with lysate only. NadCD only produced catechol (blue) from PCA (red) when in the presence of a prFMN source. Each bar is the average of three trials with error bars representing one standard deviation.

To test this hypothesis, Saro_3877 (*nadC*) and Saro_3878 (*nadD*) were amplified from the genome of *N. aromaticivorans*, cloned into expression vectors, and purified recombinant enzymes (Fig. S3) were tested for PCA decarboxylase activity *in vitro*. Previously, researchers demonstrated that a source of prFMN can be produced by overexpression of the *E.coli* prenyltransferase when this bacterium is grown under anaerobic conditions in the presence of riboflavin (FMN precursor) and prenol (as a prenyl source) (43). In this study, we used a similar procedure, based on heterologous expression of the *E. cloacae* EcdB prenyltransferase in *E. coli*, to obtain crude cell extracts that contained prFMN. We then tested purified recombinant NadCD for PCA decarboxylase activity in the presence and absence of the crude cell extracts containing the predicted prFMN cofactor. As expected, we failed to observe detectable loss of PCA or production of catechol when purified NadCD was supplied 1 mM PCA with no source of prFMN (Fig. 3). However, when the same purified NadCD was mixed with PCA and with the crude *E. coli* lysate that contained prFMN, the PCA was converted into catechol (Fig. 3). We propose that the lack of stoichiometric conversion of PCA to catechol in these longer incubation assays reflects catechol degradation via its well-known abiotic oxidation reaction (21). A control reaction in which PCA was mixed with the prFMN-containing *E. coli* lysate and no purified recombinant NadCD failed to detect any loss of PCA or production of catechol over the same incubation period (Fig. 3). From these results, we conclude that NadCD has PCA decarboxylase activity and that, like most previously studied hydroxyarylic acid decarboxylases, requires prFMN for catalysis.

**Fig 4:**
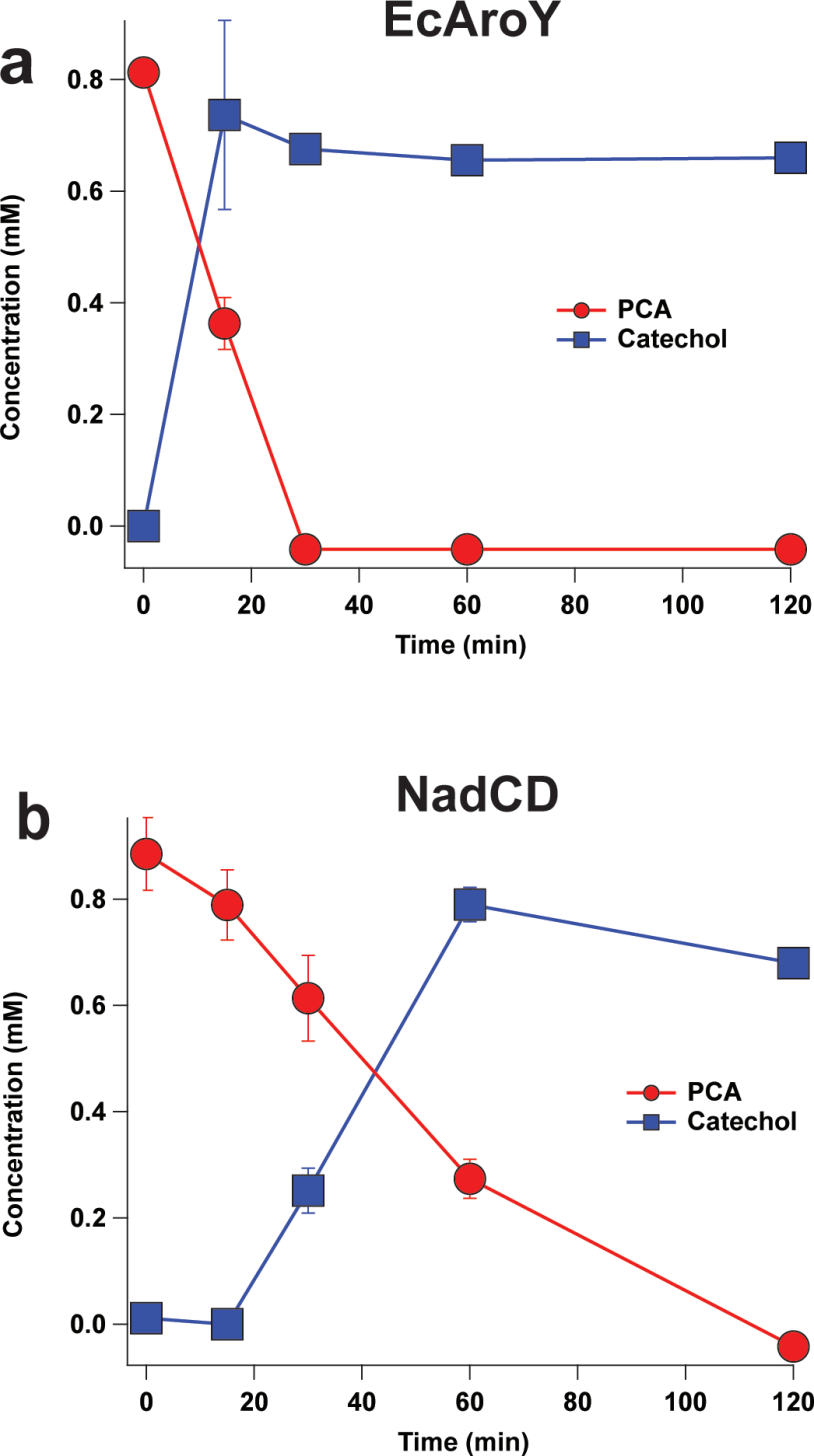
Time-dependent conversion of PCA (red) to catechol (blue) by either 100 nM EcAroY (a) or 100 nM NadCD (b) with 1 mM PCA in prFMN lysate in 50 mM HEPES, 150 mM NaCl pH 7.5. Each data point represents the average of three trials with error bars representing one standard deviation.

We also sought to compare the activity of NadCD to EcAroY, which is an extensively studied PCA decarboxylase from *E. cloacae* (38, 44). Temporal analysis of PCA decarboxylation by recombinant NadCD and EcAroY indicated that, under identical reaction conditions, EcAroY produced stoichiometric catechol from PCA within 15 min whereas 1 h was needed for NadCD to produce stoichiometric levels of catechol (Fig. 4). These results suggest that the recombinant *N. aromaticivorans* protein performs PCA decarboxylation slower than EcAroY *in vitro*. Overall, these results confirm the predicted function of *N. aromaticivorans* NadCD as a PCA decarboxylase that converts PCA to catechol. It also predicts that the NadCD decarboxylase activity was responsible for the observed PCA metabolism in the 12444_Δ*ligAB1/2* strain (Fig. 2) and that a derivative of this mutant which also lacks the *nadBCD* genes should exhibit a defect in PCA metabolism.

### Loss of both nad*BCD* and *ligAB1/2* is sufficient to accumulate extracellular PCA

To test this hypothesis, we generated a strain which lacks both *ligAB1/2* and the Saro_3873–8 gene cluster, which contains *nadBCD* (12444_PCA; Table 1). We grew the parent strain 12444, the 12444_Δ*ligAB1/2,* and 12444_PCA mutants in media containing 2 mM vanillic acid and 10 mM glucose as an additional carbon source. We found that all strains fully consumed vanillic acid but that strain 12444_PCA reproducibly grew to a lower cell density than 12444 strain or the 12444_Δ*ligAB1/2* mutant (Fig. 2). We hypothesized that the lower cell density was due to failure of 12444_PCA to assimilate the products of vanillic acid metabolism (Fig. 2a). Analysis of the extracellular metabolites of 12444_PCA revealed that the vanillic acid was consumed at the end of the incubation and that the media contained 1.4 ± 0.1 mM of PCA, representing an almost complete (78% ± 5%) recovery of extracellular PCA from vanillic acid. We propose that the less than stoichiometric recovery of PCA in the media at the end of the incubation is due to the previously reported abiotic oxidation of PCA (45–47). From these results, we concluded that NadBCD contributes to PCA metabolism of 12444 *N*. *aromaticivorans*. We further propose that the combined deletion of the *nadBCD* and *ligAB1/2* genes generates a *N. aromaticivorans* strain (12444_PCA) that excretes PCA because it has a defect in assimilation of this intermediate in aromatic metabolism.

### Converting PCA to catechol

Extracellular accumulation of PCA by strain 12444_PCA predicts that there are no other major pathways for PCA metabolism in *N. aromaticivorans*. This finding enabled us to use 12444_PCA as a platform strain to test if we could divert the PCA derived from H and G family aromatics toward *cc*MA production. Since bacterial production of *cc*MA often proceeds through the intradiol aromatic ring cleavage of catechol (Fig. 1) (5, 6, 22), we sought to divert PCA toward this ring-opening pathway. However, conversion of PCA to catechol has been observed as a metabolic bottleneck in other engineered bacterial strains (5, 6). Since the activity of the *N. aromaticivorans* NadCD enzyme was lower than that of the well-studied PCA decarboxylase EcAroY, we generated two strains which contained either *nadBCD* (LigAB1_NaDec) or *ecdB/ecaroY/ecdD* (LigAB1_EcDec) in the *ligAB1* (Saro_2812–13) locus of 12444_PCA. We chose to place genes encoding either of the PCA decarboxylase genes in the *ligAB1* locus as this locus is highly transcribed when *N. aromaticivorans* is grown in the presence of G family aromatic compounds (32). Based on the activity of these two decarboxylases *in vitro*, we predicted that insertion of these PCA decarboxylase genes into the *ligAB1* locus of the 12444_PCA strain should allow conversion of PCA to catechol *in vivo* and reduce or block extracellular PCA accumulation.

**Fig 5:**
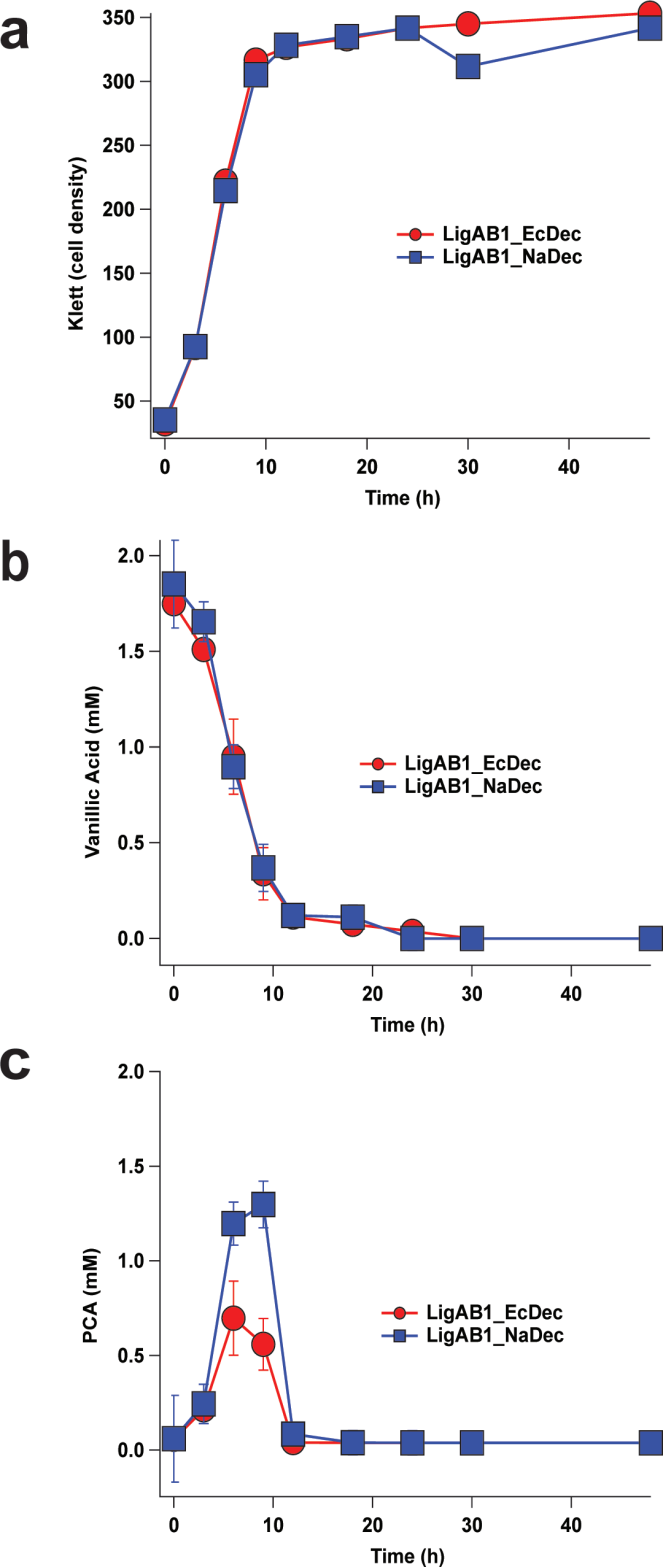
Cell density (a) and extracellular metabolite concentration of vanillic acid (b) and PCA (c) of LigAB1_EcDec (red) and LigAB1_NaDec (blue). Cultures were grown with 2 mM vanillic acid and 10 mM glucose, and metabolite concentrations were analyzed by liquid chromatography mass spectroscopy. The data points are the average of three trials with error bars representing one standard deviation.

To test this hypothesis, we grew strains LigAB1_EcDec and LigAB1_NaDec in media containing 2 mM vanillic acid and 10 mM glucose as an auxiliary carbon source. We found that both the LigAB1_EcDec and LigAB1_NaDec strains reached similar final cell densities and completely consumed vanillic acid within 12 h (Fig. 5). We observed transient accumulation of extracellular PCA in each strain, reaching a maximum of 1.4 ± 0.1 mM in the LigAB1_NaDec at 10 h and 0.8 ± 0.2 mM at 6 h for LigAB1_EcDec (Fig. 5). However, extracellular PCA was undetectable in both cultures after 12 h (Fig. S6). These results indicate that placement of either the Ec or Na PCA decarboxylase genes at the *ligAB1* locus of the 12444_PCA strain restored the strain’s ability to assimilate this pathway intermediate. In comparison to the 12444_Δ*ligAB1/2* strain, which has the genes for *nadBCD* in its native locus, both of the above strains consume PCA faster with full consumption of this pathway intermediate within 12 h compared to 24 h. This is consistent with the observation of higher transcript levels from the *ligAB1* locus versus the *nadBCD* locus when cells are grown in the presence of G family aromatics (32).

### *N. aromaticivorans* genes predicted to be involved in catechol metabolism

While both LigAB1_NaDec and LigAB1_EcDec were able to metabolize PCA, neither of these strains accumulated detectable levels of extracellular catechol or *cc*MA (Fig. S6). To explain this observation, we propose that these strains contain one or more previously undescribed pathways for catechol assimilation into cellular material. Therefore, we sought to identify potential enzymes involved in catechol metabolism in *N. aromaticivorans*.

Bacterial catechol catabolism can be initiated via extradiol cleavage by a 2,3 dioxygenase, XylE, producing 2-hydroxymuconate semialdehyde or through intradiol cleavage by a 1,2-catechol dioxygenase, CatA, to generate *cc*MA (Fig. 1). To predict the potential *N. aromaticivorans* pathways for catechol metabolism, we analyzed its genome for homologs of genes that encode enzymes that function in the extradiol and intradiol pathways. This analysis predicted the presence of *catBCA and xylEGHIJKQ* (25) transcription units that encode *N. aromaticivorans* proteins with >40% amino acid identity to known enzymes in the intradiol and extradiol cleavage pathways, respectively (Tables S4 and S5), suggesting that *N. aromaticivorans* can potentially metabolize catechol by both pathways.

**Fig 6:**
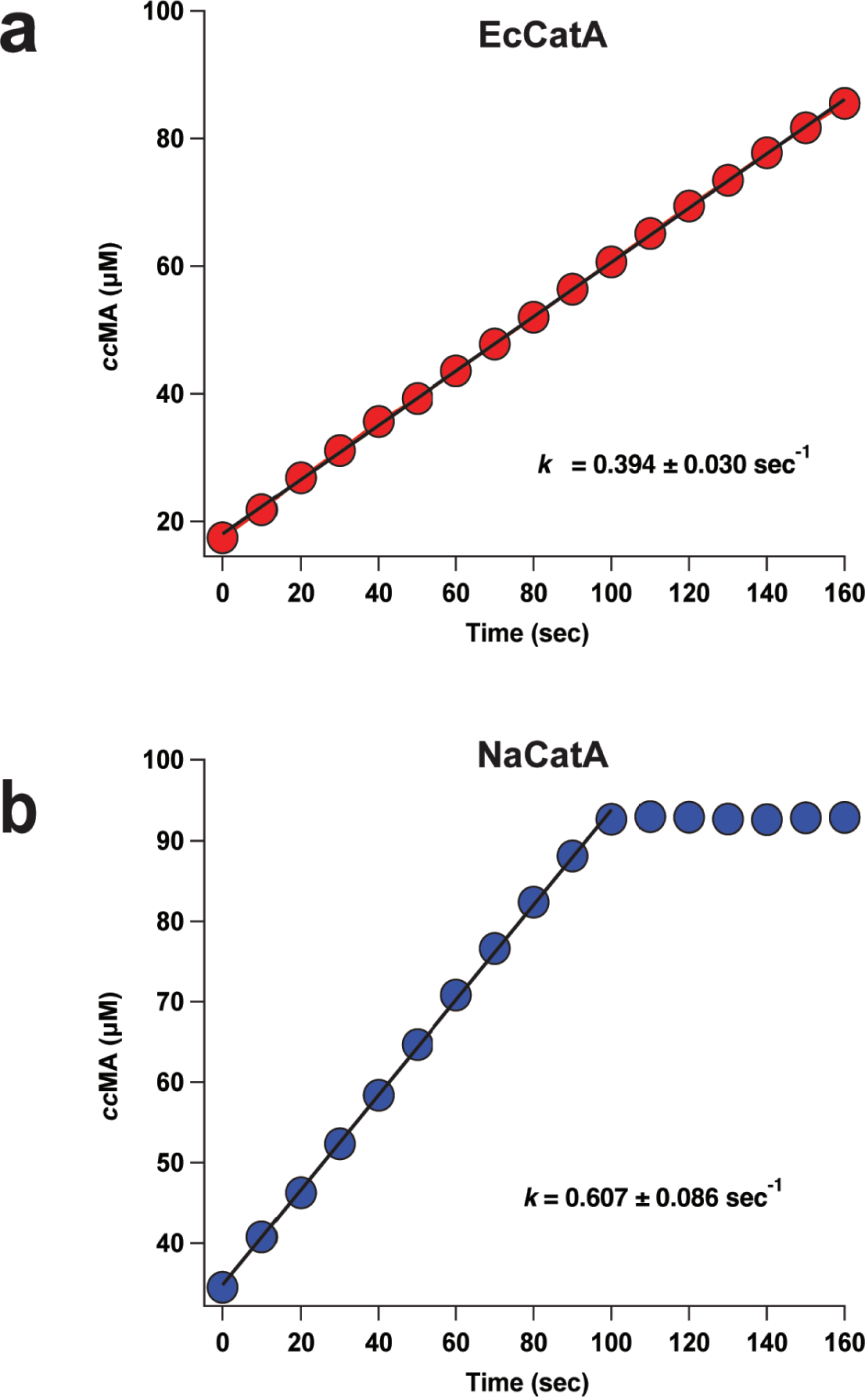
Representative time courses of the formation of *cc*MA with either EcCatA (red) or NaCatA (blue). Both reactions were performed in 50 mM HEPES 150 mM NaCl pH 7.5 with either 0.5 µM EcCatA (a) or NaCatA (b) and initiated with 100 μM catechol. The formation of *cc*MA was monitored by UV/vis absorption spectroscopy at λ_260-nm_ and the data points were best fit to a linear equation ([*cc*MA] = kt + [*cc*MA]_0_). The resulting fit is shown as the black line. The inset shows the average rate after three trials with error represented by one standard deviation above and below the mean.

Of these two potential catechol cleavage pathways, only CatA is predicted to generate *cc*MA. Unlike some other aromatic metabolizing bacteria (48), the *N. aromaticivorans* genome is not predicted to contain a second copy of a gene that encodes a protein with significant amino acid sequence identity to known CatA enzymes (Table S5). Thus, we sought to test the activity of the predicted, but previously uncharacterized *N. aromaticivorans* CatA enzyme (NaCatA), and to compare it to the CatA of *E. cloacae* (EcCatA). To do this, the catechol 1,2 dioxygenase activity of purified recombinant NaCatA and EcCatA proteins (Fig. S3) was monitored by following the absorbance at 260 nm corresponding to *cc*MA by UV/visible absorption spectroscopy (49). Under identical assay conditions, both EcCatA and NaCatA produced stoichiometric (Fig. S1) amounts of *cc*MA and the data best fit to a linear equation yielding zeroth order rate constants on the same order of magnitude of each other (Fig. 6). From this, we conclude that NaCatA catalyzes the intradiol cleavage of catechol to produce *cc*MA *in vitro* and exhibits a similar rate of activity as EcCatA under these assay conditions.

### Engineering a *N. aromaticivorans* strain to divert catechol to *cc*MA

Based on the genomic, bioinformatic, and *in vitro* analysis of *N. aromaticivorans* enzymes that are predicted to be involved in catechol metabolism, we reasoned that several genetic modifications were needed to engineer a strain that accumulated extracellular *cc*MA from pathway intermediates like PCA. First, the existence of a *catBCA* operon for intradiol cleavage of catechol predicted deletion of *catBC* genes would be needed in order to block metabolism of the *cc*MA generated by CatA activity. To do this, we generated Δ*catBC* derivatives of strains LigAB1_NaDec and LigAB1_EcDec, which produced the strains, NaDec_cat and EcDec_cat, respectively (Table 1). We also reasoned that inactivation of the *xylE*-dependent pathway for extradiol cleavage of catechol would be needed to divert catechol through the intradiol CatA-dependent pathway. Therefore, we replaced the native *N. aromaticivorans xylE* with either *eccatA* or the *nacatA* producing EcDec_*cc*MA or NaDec_*cc*MA, respectively (Table 1). We inserted *catA* into the native *xylE* locus since the transcript abundance of the *xyl*E gene in *N. aromaticivorans* is higher than *catA* transcript levels when wild-type cells are grown in the presence of aromatics (Fig. S2) (32). We reasoned that placement of *eccatA* or *nacatA* at the *xylE* locus should help ensure that these engineered strains were expressing sufficient levels of CatA to completely metabolize catechol to *cc*MA. We chose to generate strains expressing either EcCatA or NaCatA from the same locus in order to test whether the two versions of CatA led to production of significantly different levels of extracellular *cc*MA.

**Fig 7:**
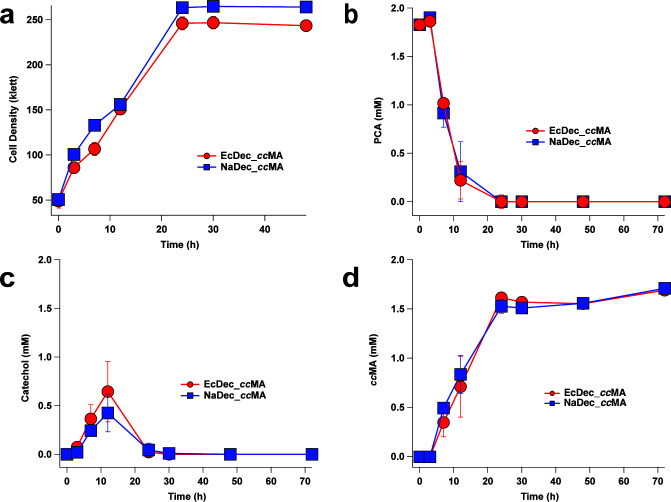
Cell density (a) of EcDec_*cc*MA (red) and NaDec_*cc*MA (blue) and extracellular metabolite concentration of PCA (b), catechol (c), and *cc*MA (d). Cultures were grown in a shake flask with 2 mM PCA and 10 mM glucose. Metabolite concentrations were analyzed by liquid chromatography mass spectroscopy. Each data point represents the average of three trials with error bars representing one standard deviation.

To test the impact of these genomic alterations on aromatic metabolism, we evaluated the ability of EcDec_*cc*MA and NaDec_*cc*MA to convert PCA into *cc*MA by growing these strains in media containing 2 mM PCA and 10 mM glucose as an auxiliary carbon source. Both strains exhibited transient accumulation of catechol and completely converted PCA to *cc*MA within 30 h with *cc*MA yields of 100 ± 5% for NaDec_*cc*MA or 97 ± 3% for EcDec_*cc*MA (Fig. 7 and Fig. S8). These results illustrate that these alterations to the genome of *N. aromaticivorans* allowed for funneling of PCA to the catechol intradiol branch and subsequent *cc*MA production. This finding provides new evidence in support of the hypothesis that *N. aromaticivorans* can be used as a chassis for the metabolic conversion of G and H aromatics into *cc*MA.

### Synthesis of *cc*MA from aromatics in poplar APL

To further evaluate the use of *N. aromaticivorans* as a chassis for *cc*MA production, we tested the ability of the NaDec_*cc*MA and EcDec_*cc*MA strains to produce this compound from biomass-derived aromatics. It is known that transgenic plants expressing the quinate and shikimate utilization B (*qsuB*) gene increase the accumulation of aromatics, notably PCA, found in biomass (50). Previously, we have shown that these QsuB transgenic poplar plants can be used as a source of aromatics for the conversion of biomass aromatics to PDC (51). Thus, we tested the ability of the NaDec_*cc*MA and EcDec_*cc*MA strains to produce *cc*MA from aromatics derived from a transgenic poplar QsuB plant.

**Fig 8:**
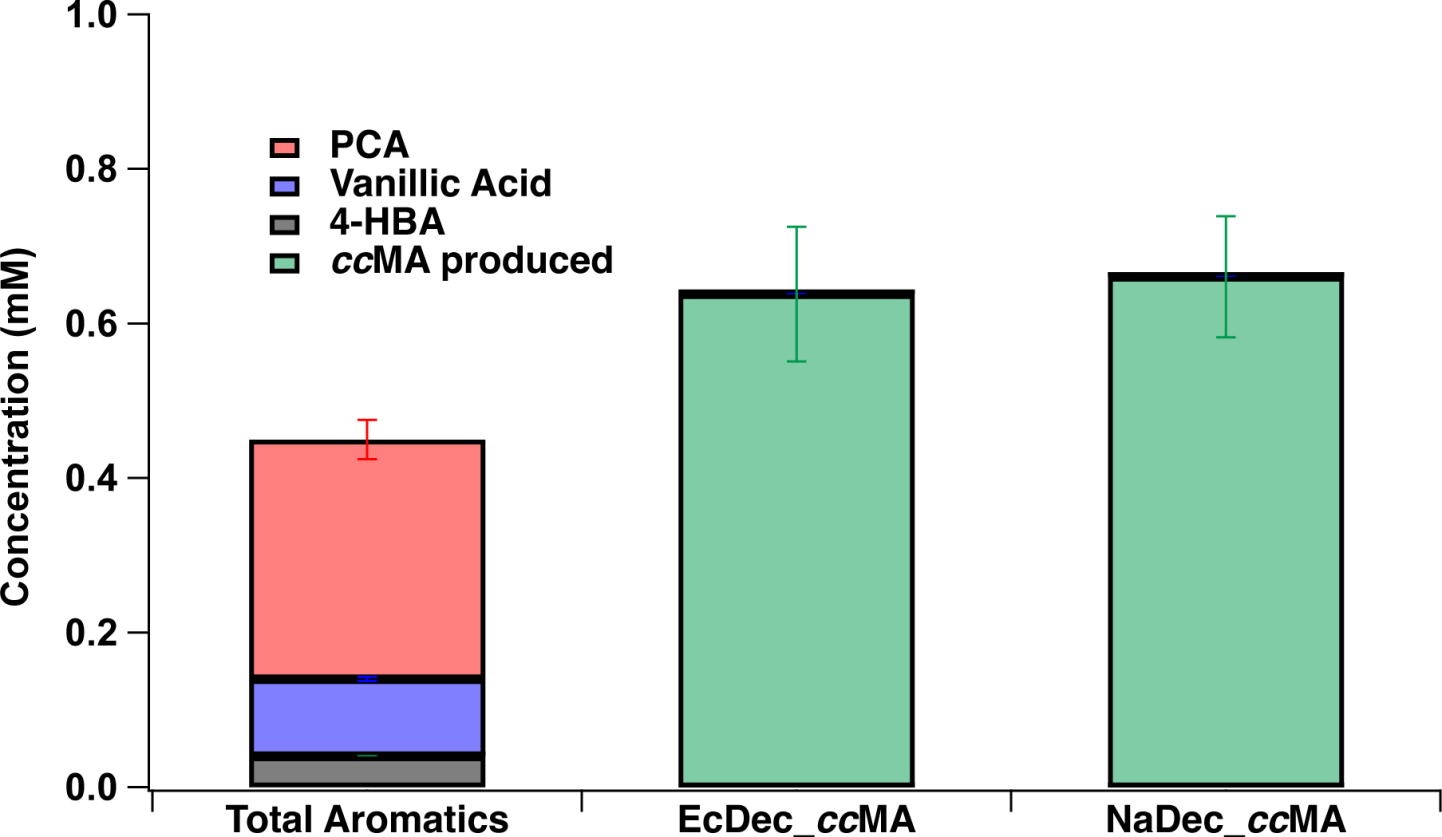
Conversion of Qsub APL into *cc*MA with either EcDec_*cc*MA or NaDec_*cc*MA *N. aromaticivorans* strains. The first bar represents the total concentration of the major free and glycosylated aromatics identified from Qsub APL with PCA (red), vanillic acid (blue), and 4-hydroxybenzoic acid (gray). Next, the bars represent the concentration of *cc*MA produced (green) from either the EcDec_*cc*MA or NaDec_*cc*MA strains after 48h incubation with Qsub APL. Each bar is the average of three trials with error bars representing one standard deviation.

An aqueous solution containing both phenolic monomers and glycosylated forms of PCA and vanillic acid was obtained from QsuB poplar biomass using a mild alkaline pretreatment that cleaves ester linkages (Fig. S9) (51). After acid treatment of the APL to release the glycosylated phenolic compounds (51), the major identifiable phenolics in this material were PCA, vanillic acid, and 4-HBA with trace amounts of ferulic acid and 4-coumaric acid (Table S11). It is known that *N. aromaticivorans* is able to metabolize both free and glycosylated phenolics (51), so the concentration of known phenolics derived from the Qsub poplar biomass was calculated to include the concentration of the glycosylated phenolics (51). This calculation showed that we obtained 0.42 ± 0.03 mM of total aromatics in the Qsub APL with PCA accounting approximately 60% of the total aromatics (Fig. 8 and Fig. S9).

To test for microbial production of *cc*MA from the aromatics in QsuB poplar APL, we added glucose as an auxiliary carbon source and ammonium sulfate as a nitrogen source to cultures of EcDec_*cc*MA or NaDec_*cc*MA. After 48 h, both strains produced *cc*MA from QsuB poplar APL with calculated yields of 157 ± 26% for EcDec_*cc*MA and 163 ± 25% NaDec_*cc*MA (Fig. 8). After 48 h of incubation, we did not observe any 4-HBA or PCA in the medium and only trace amounts of vanillic acid (Fig. S9), which indicates that these APL aromatics were metabolized and possibly converted to *cc*MA. From this, we conclude that the EcDec_*cc*MA and NaDec_*cc*MA strains were both capable of production of *cc*MA from Qsub APL poplar. Possible explanations for the greater than 100% yield of *cc*MA from components of the Qsub poplar APL are provided in the discussion.

## DISCUSSION

Lignin is the second most abundant renewable polymer on Earth (8) and represents a potential source of phenolics for conversion into industrial chemicals and materials (12). Despite this, the heterogeneity of aromatic monomers and their inter-subunit linkages has presented challenges in producing sources of valuable chemicals from this abundant resource. In recent years, the ability of some microbes to funnel a diverse set of aromatics to common intermediates has catalyzed interest in using genome-enabled strain engineering to generate one or more valuable compounds from these phenolic mixtures (7, 15, 36). This study investigated the utility of the aromatic metabolizing bacterium *N. aromaticivorans* as a chassis for *cc*MA production from biomass aromatics by combining an expanded knowledge base of its aromatic metabolic pathways with metabolic engineering.

Microbial production of *cc*MA from glucose has been reported in bacteria that either lack or have a limited ability to metabolize the mixed aromatics that are abundant in plant biomass (2, 52). Many efforts to produce *cc*MA from aromatics have been limited by the accumulation of pathway intermediates (6, 20, 22, 23). We chose *N. aromaticivorans* as a potential host for *cc*MA production as it has the native ability to metabolize the major aromatic monomers found in plant cells walls, to transport and cleave low molecular weight aromatic oligomers with different inter-subunit linkages, and to convert modified phenolics that are formed by some methods of biomass or lignin deconstruction (11, 29, 53). Below, we summarize how we combined bioinformatics, enzymology and the genetic tractability of *N. aromaticivorans* to gain knowledge about its pathways for aromatic metabolism and use this information to engineer strains that produce *cc*MA from aromatics present in biomass.

### Diverting PCA to catechol

Previous studies that engineered *N. aromaticivorans* to produce PDC from aromatics suggested that the extradiol cleavage of PCA by 4,5 PCA dioxygenase homologs (LigAB1 and LigAB2) represented a major route for metabolism of this pathway intermediate (30, 31). The results of these studies also predicted the possibility of another minor route for PCA metabolism since yields of PDC were significantly less than 100% when a PDC-producing strain was grown in the presence of PCA (30, 31). Furthermore, work with a derivative of the PDC producing strain that had deletions of both the *ligAB1* and *ligAB2* gene sets found that, when grown with vanillic acid, the cells accumulated only ~50% of expected PCA and failed to accumulate PDC (31). Combined, these results suggest that LigAB1 and LigAB2 both play a significant role in PCA metabolism but that another PCA metabolic pathway exists. In this study, we identified a previously uncharacterized PCA decarboxylase as the enzyme that is responsible for consumption of PCA in cells that lack the two 4,5 PCA dioxygenase homologs (LigAB1 and LigAB2).

As predicted from previous work (30), we found that inactivation of both LigAB1 and LigAB2 in the 12444_Δ*ligAB1/2* strain resulted in only transient PCA accumulation from vanillic acid (Fig. 2), leading us to conclude that this mutant was able to consume PCA by another catabolic pathway. Analysis of both the *N. aromaticivorans* genome sequence and of the transcript levels when cells are grown in the presence of G family aromatics suggested that a previously uncharacterized PCA decarboxylase could be converting PCA to catechol when genes encoding both LigAB homologs were deleted. In addition, a published analysis of a genome-wide transposon insertion library suggested a connection between PCA and catechol metabolism in *N. aromaticivorans* (25). Prior to this work, there was no experimental evidence for the function of a PCA decarboxylase in *N. aromaticivorans*. Our genetic data and *in vitro* enzyme assays of the previously uncharacterized *N. aromaticivorans* NadCD led us to propose that NadCD is the PCA decarboxylase that is responsible for conversion of PCA to catechol (Fig. 1). As predicted by this hypothesis, we found that deletion of the genes encoding NadCB in the 12444_Δ*ligAB1/2* strain resulted in the accumulation of extracellular PCA. We think it is unlikely that the less than 100% PCA recovery (78 ± 5%) in this strain reflects the function of another unknown PCA catabolic pathway in *N. aromaticivorans*. In support of this hypothesis, we could not identify genes that encode proteins with >25% amino acid identity to the intradiol 3,4 or to the extradiol 2,3 PCA dioxygenases found in other reported PCA catabolic pathways (34–36). Instead, our data lead us to propose that a heretofore uncharacterized *N. aromaticivorans* decarboxylase (NadCD) is the route for conversion of PCA to catechol, and that the less than 100% recovery of PCA reflects the well-known abiotic oxidation of this compound (45–47).

The prediction that *N. aromaticivorans* contains a PCA decarboxylase (NadCD) was not expected as there are only a few characterized homologs of this enzyme in aromatic-metabolizing bacteria (38, 44, 54). Indeed, while most metabolic engineering strategies for *cc*MA production using aromatic-metabolizing bacteria take advantage of the intradiol cleavage pathway of aromatic metabolism for *cc*MA production, they often utilize a PCA decarboxylase from another bacterium (often *E. cloacae* or *K. pneumoniae*) to convert PCA into catechol (4, 20, 55). In addition, the conversion of PCA to catechol is often a bottleneck in *cc*MA production; one that has been circumvented in *P. putida* KT2440 by using a foreign promoter to increase expression of a foreign PCA decarboxylase that is comprised of the EcdB/EcAroY/EcdD proteins. For these reasons, we sought to compare the PCA decarboxylase activity of NadCD to that of the commonly used EcAroY/EcdD decarboxylase.

Our *in vitro* analysis of recombinant NadCD and EcAroY showed that under identical conditions, the *N. aromaticivorans* enzyme was active, albeit slower, than the *E. cloacae* homolog. The lower activity of NadCD compared to EcAroY *in vitro* suggested that the conversion of PCA to catechol *in vivo* could be slower for engineered strains that depend on the native PCA decarboxylase. Indeed, the LigAB1_NaDec strain showed a higher transient extracellular level of PCA *in vivo* than the strain containing genes encoding the *E. cloacae* decarboxylase at the same locus (LigAB1_EcDec). Nevertheless, only transient accumulation of PCA was found when either LigAB1_EcDec or LigAB1_NaDec are grown with vanillic acid, indicating active PCA decarboxylation in either strain and full consumption of PCA by the end of the experiment. Future experiments to elucidate the structure of the newly discovered *N. aromaticivorans* PCA decarboxylase could inform protein and strain engineering studies aimed at improving the rates of PCA conversion to catechol, which is reported to be a bottleneck for *cc*MA production in other microbes (5, 6).

This study also demonstrated that the insertion of the *nadBCD* genes into the *ligAB1* locus resulted in faster PCA consumption as compared to the 12444_Δ*ligAB1/2* strain, which has *nadBCD* in its native locus. The faster PCA consumption observed in the LigAB1_NaDec strain is consistent with the previous transcript analysis of *N. aromaticivorans* that showed higher abundance of *ligAB1* transcripts than those from *nadBCD* when cells are grown in the presence of G family aromatics (32). These results demonstrated that placement of either *naBCD* or *ecdB/ecaroY/ecdD* into the *ligAB1* locus provided sufficient decarboxylase activity for PCA consumption. From this, we conclude that the predicted PCA decarboxylase transcript levels in the LigAB1_NaDec strain are higher than those which express the PCA decarboxylase only from its native locus and contributes to faster PCA decarboxylation by this strain *in vivo*.

### Diverting catechol to *cc*MA

Previous analysis of *N. aromaticivorans* indicated high transcript levels of the catechol 2,3-dioxygenase (*xylE*) when cells are grown in the presence of PCA or one of several G family aromatics (32). These results suggest that catechol can be metabolized through the extradiol pathway (Fig. 1). However, evidence was lacking for the presence or function of a CatA homolog in the intradiol cleavage pathway of catechol to *cc*MA in this bacterium. While the *N. aromaticivorans* genome encodes a protein with amino acid sequence identity to other CatA enzymes (Table S5)*, nacatA* transcripts are lower than those encoding enzymes of other known aromatic metabolizing enzymes when cells are grown in the presence of G family aromatics (32). There is also no published evidence for metabolism of catechol via the intradiol branch in *N. aromaticivorans*. Therefore, to generate additional knowledge of *N. aromaticivorans* aromatic metabolism, we tested a purified recombinant NaCatA for catechol 1,2-dioxygenase activity.

Our *in vitro* results indicated that recombinant NaCatA was active for catechol 1,2-dioxygenase activity and that it has comparable activity to recombinant EcCatA enzyme. Other catechol 1,2-dioxygenases typically follow Michaelis-Menten kinetics (56), so the zeroth order plot obtained with either CatA from *N. aromaticivorans* or *E. cloacae* suggests substrate saturation by catechol of both enzymes. In addition, our results suggest that *N. aromaticivorans* encodes a catechol 1,2-dioxygenase that is capable of converting catechol to *cc*MA. Overall, the results of these experiments are the first report that *N. aromaticivorans* has the ability to metabolize aromatics via the intradiol branch of catechol catabolism. However, additional kinetic analyses are needed to test the hypothesis that both enzymes exhibit typical Michaelis-Menten kinetics.

### *cc*MA production from biomass aromatics by *N. aromaticivorans*

Comparison between the transcript abundance of *catA* and *xylE* could be interpreted to predict that the extradiol (*xylE*)-dependent pathway is the major pathway for catechol catabolism in *N. aromaticivorans*. Indeed, the relatively low *catA* transcript abundance when cells were grown in the presence of aromatics suggested there might be little to no flux through this intradiol CatA-dependent pathway in these cultures. Additionally, the presence of only one gene encoding a protein with amino acid sequence similarity to known CatA enzymes suggested there was a potential bottleneck in the conversion of catechol to *cc*MA when relying on the expression of *catA* from its native locus. Therefore, the genes for *nacatA* and *eccatA* were separately placed into the *xylE* locus, a region that is highly transcribed when cells are grown in the presence of aromatics (32). We also inactivated *xylE* and *catBC* in order to generate a strain which is predicted to only metabolize catechol via the intradiol pathway and be unable to metabolize *cc*MA. This generated two *N. aromaticivorans* strains to compare for *cc*MA accumulation using genes derived from either *N. aromaticivorans* (NaDec_*cc*MA) or *E. cloacae* (EcDec_*cc*MA). As predicted by the similar rates observed *in vitro* for EcCatA or NaCatA, when cells were grown with PCA, both strains accumulated minimal amounts of catechol and produced stoichiometric *cc*MA at a similar rate. Stoichiometric conversion of *cc*MA from PCA indicated that the knowledge gained from these experiments on the aromatic metabolism of *N. aromaticivorans* was successfully implemented to direct PCA metabolism to the catechol intradiol pathway using either native genes or genes derived from *E. cloacae*.

To date, strains tested for *cc*MA production from crude biomass aromatics produced *cc*MA yields ranging from 5% to 100%, with most strains producing less than 50% yields of *cc*MA from deconstructed lignin (20). To test the feasibility of our engineered *N. aromaticivorans* strains to produce *cc*MA from biomass aromatics, we chose to use QsuB poplar biomass (50) because (i) our strains were engineered to use PCA as a precursor for *cc*MA production and (ii) our results showed they were capable of stoichiometric conversion of PCA to *cc*MA. Therefore, we predicted that when our strains were grown in batch cultures with this APL source, we would get high yields of *cc*MA. Indeed, at the end of the batch culture period, liquid chromatography mass spectroscopy (LCMS) analysis showed one major peak corresponding to *cc*MA in the extracellular media from the NaDec_*cc*MA and EcDec_*cc*MA cultures (Fig. S10). The aromatics that were quantified in the APL included PCA, vanillic acid, and 4-HBA (Fig. S9), with the majority of PCA and vanillic acid found in glycosylated forms. All the PCA and 4-HBA were transformed during incubation, with only trace amounts of vanillic acid remaining after the incubation. Umana et al. demonstrated that the glycosylated forms of PCA and vanillic acid are degraded by *N. aromaticivorans*, and as PCA is an intermediate in the degradation of vanillic acid and 4-HBA, this observation leads us to conclude that these strains were producing *cc*MA from all the aromatics quantified in the biomass APL (51). However, the greater than 100% *cc*MA yield in the NaDec_*cc*MA and EcDec_*cc*MA cultures grown in the presence of APL suggests that both of these engineered strains are capable of metabolizing other unidentified aromatics present in the APL.

Furthermore, since the mild alkaline pretreatment process used to generate the APL cleaves the ester bonds and liberates soluble metabolites without breaking down the lignin backbone (51), it is likely that these unidentified aromatics are not oligomeric forms of deconstructed lignin. Nevertheless, the diversity of aromatic catabolic pathways available in *N. aromaticivorans* could be a valuable characteristic of *N. aromaticivorans* since lignocellulosic biomass deconstruction methods that are more aggressive than the mild alkaline pretreatment used in this study would be expected to produce a range of aromatic monomers, dimers, and oligomers, as well as sugar conjugates and other organic materials that could be used to support growth of engineered strains.

Overall, this work increases our knowledge on the diversity of aromatic metabolic routes available in *N. aromaticivorans*. We identified unreported *N. aromaticivorans* metabolic pathways that are involved in the conversion of PCA to *cc*MA. *In vitro* characterization of newly identified PCA decarboxylase (NadCD) and catechol 1,2-dioxygenase (NaCatA) enzymes predicted the existence of formerly unknown metabolic routes for aromatic metabolism. We confirmed the function of these metabolic pathways through creation of defined mutants that demonstrated a new route for PCA catabolism to catechol, as well as the function of an intradiol pathway for catechol metabolism in *N. aromaticivorans*. The existence of a native PCA decarboxylase in *N. aromaticivorans* is somewhat unique in comparison to other reported *cc*MA producing hosts which do not naturally possess a PCA decarboxylase capable of converting PCA to catechol (20). The pathways for PCA catabolism in *N. aromaticivorans* are also different in comparison to other sphingomonads such as *Sphingobium* sp. SYK-6, since we were unable to identify genes in this well-studied aromatic metabolizing bacterium that encode proteins with significant amino acid sequence identity to known PCA decarboxylases (37). In addition, while we could identify a *Sphingobium* sp. SYK-6 *catA* homolog that encoded a protein with ~40% amino acid sequence identity to *E. cloacae* CatA, the genome is not predicted to encode proteins with amino acid sequence identity to the typical CatBC enzymes of the catechol intradiol catabolic pathway. These observations increase our knowledge of the number and diversity of *N. aromaticivorans* aromatic catabolic pathways and further highlight the potential of this bacterium as a host for converting aromatics into commodity chemicals.

Our biochemical and genetic characterization of previously uncharacterized *N. aromaticivorans* gene products allowed for the generation of an engineered *cc*MA-producing microbe that is completely derived from native genes and transcriptional units. The use of native enzymes is potentially advantageous as it likely bypasses problems associated with folding or stability of foreign proteins, availability of required cofactors, and the accumulation of unusual intermediates that are part of a pathway that is not normally used by the host. In addition, we found that the *N. aromaticivorans* PCA decarboxylase and CatA proteins have activity that is comparable to that of well-studied enzymes from other hosts that have been used to build other *cc*MA production strains. Indeed, comparison of NaDec_*cc*MA and EcDec_*cc*MA demonstrated that both strains produced *cc*MA at similar yields and rates from either pure aromatics or biomass-derived aromatics. These results suggest that it will be possible to further engineer *N. aromaticivorans* strains for improved *cc*MA productivity using solely native genes.

Technoeconomic analyses of the conversion of renewable aromatics by microbes predict that strains need to generate products at high titers, rates, and yields from the different aromatic mixtures that are found in biomass (15, 57, 58). This work demonstrated the ability to create a *N. aromaticivorans* strain that stoichiometrically converts the aromatics in Qsub poplar APL into *cc*MA when cells are grown in batch cultures. The type and concentrations of aromatics in lignin-derived streams will vary depending on the energy crop used and on the chemical methods applied for biomass deconstruction and lignin depolymerization (8, 15, 59). Future studies could aim at determining *cc*MA production rates, titers, and yields from different lignin streams, which will contain a mixture of native and chemically modified aromatics (60, 61). Data from these studies can inform future efforts to use or modify existing strains of *P. putida* KT2440, *N. aromaticivorans* DSM12444, or other bacteria for production of *cc*MA and/or other products from one or more concentrated biomass aromatic solutions at industrial scale. As it is unlikely that there is only one perfect choice organism for every lignin stream, these studies will provide a broad scope of microorganisms that could be tailored to specific lignin streams for industrial scale generation of bioproducts.

In conclusion, our findings have expanded the knowledge of aromatic catabolic pathways in *N. aromaticivorans* and demonstrated the utility of this bacterium as a chassis for *cc*MA production from phenolic mixtures derived from lignocellulosic biomass. Our studies provide a proof of concept for stoichiometric *cc*MA production from *N. aromaticivorans* and generate a host that can be used for future studies to optimize *cc*MA production rates, titers, and yields in bioreactors. The new findings reported in this paper also illustrate the value of the genetic and metabolic tractability of the abundant aromatic catabolism pathways in *N. aromaticivorans* as engineering of these *cc*MA-producing strains did not require the use of synthetic promoters and additional genomic alterations to produce stoichiometric yields of *cc*MA from deconstructed biomass. Overall, this work provides new insights in the aromatic metabolism of *N. aromaticivorans* and highlights the potential for using this bacterium as a host for producing additional valuable products from biomass aromatics.

## MATERIALS AND METHODS

### Bacterial strains, growth media, and culturing conditions

*N. aromaticivorans* 12444Δ1879 (30) (called 12444 in Table 1) is a derivative of wild-type strain DSM 12444 in which a putative *sacB* gene (Saro_1879) was deleted to create a strain amenable to genomic modifications using a variant of the pK18*mobsacB* plasmid (62) that contains both kanamycin resistance and *sacB*. Plasmids for cloning were constructed with the NEBuilder HiFi DNA Assembly Master Mix (New England Biolabs; Ipswich, MA), as described in the supplementary material SI. A complete list of the mutant strains and a list of the primers used to generate these mutant strains are shown in Table 1 and Table S2.

*E. coli* DH5α cells were used in all plasmid preparations and either *E. coli* S17 or *E. coli* WM6026 were used as a conjugal donor for mobilization of DNA into *N. aromaticivorans*. Procedures for conjugation and modifying the *N. aromaticivorans* genome via homologous recombination are found in the supplementary material SI. All *E. coli* strains were grown in Lysogeny Broth (LB) media containing 50 mg/L kanamycin or 0.3 mM diaminopimelic acid (DAP) when necessary. All *N. aromaticivorans* strains were grown in standard mineral base (SMB) minimal media (63) and supplemented with 10 mM glucose and an additional aromatic when specified. For genomic modifications of *N. aromaticivorans,* the media was either supplemented with 50 mg/L of kanamycin or with 10% sucrose (100 g/L).

### *N. aromaticivorans* growth experiments

Each growth experiment was performed in triplicate. Starter cultures of the *N. aromaticivorans* strains were grown aerobically (~18 h) in 5 mL of SMB media supplemented with 10 mM glucose, in 18 × 150 mm culture tubes at 30°C. The cells were then diluted by 1:1 and regrown to mid-exponential growth phase. The SMB vanillic acid and SMB PCA solutions were prepared fresh by dissolving either 34 mg of vanillic acid or 30 mg of PCA into 100 mL of SMB, which was then passed through a sterile 0.22 µm filter. A 1:10 dilution was performed by adding 1.2 mL of starter culture into a 125 mL Erlenmeyer flask equipped with a side arm that contained 12 mL of SMB media with the specified carbon source. The cell density was measured at various time points using a a Klett-Summerson photoelectric colorimeter with a red filter (30). Aliquots of culture samples (0.2 mL) were removed at indicated time points and filtered (30) prior to storage at 4°C. LCMS analysis was performed on the day of the last time point.

### Heterologous protein expression and purification

Genes Saro_3877–78 and Saro_3830 were amplified via PCR from the *N. aromaticivorans* genome and independently cloned into the pVP302K plasmid, which contains an 8× His tag (31). *EcaroY/D* was PCR amplified from the ΔligAB1:EcDec_pK18mobsacB plasmid, and *eccatA* was PCR amplified out of a pUC57 plasmid synthesized by Genscript. Both genes were independently cloned into the pVP302K plasmid. The list of primers used to generate these protein expression plasmids and corresponding plasmids is shown in Table S1. Purified plasmid was then transformed into *E. coli* B834 containing the pRARE2 plasmid (31). Identical methods for heterologous protein expression and purification were performed and detailed below.

For protein expression, a single colony was used to inoculate a 20 mL starter culture of cells grown in LB media containing kanamycin (50 mg/L) and chloramphenicol (20 mg/L) in a 125 mL Erlenmeyer flask that was shaken at 200 rpm overnight (~18 h) at 37°C. Next, the entire 20 mL starter culture was used to inoculate a 2 L Erlenmeyer flask containing 500 mL of Terrific Broth (TB)(64) media containing kanamycin (50 mg/L) and chloramphenicol (20 mg/L). The 500 mL culture was shaken at 200 rpm at 37°C for 4 to 5 h until reaching an optical density (OD), OD_6oo_, of ~0.7. Once the cells reached an OD_6oo_ of 0.7, protein expression was induced with the addition of isopropyl β-D-1-thiogalactopyranoside (IPTG, 0.3 mM final concentration). For expression of the 1,2- catechol dioxygenase proteins, the media also included Fe(II)SO_4_ at a final concentration of 0.160 mM at the time of induction. Induction was allowed to proceed overnight (~18 h) at 20°C for both the PCA decarboxylase and the 1,2 catechol dioxygenase cultures. After induction, cells were harvested by centrifugation and suspended in the resuspension buffer which contains 50 mM HEPES (2-[4-(2-hydroxyethyl)piperazin-1-yl]ethane-1-sulfonic acid), 150 mM NaCl, and 0.1% Triton X-100 at pH 7.5. The cells were then lysed by sonication and clarified by centrifugation at 20,000 rpm for 30 min. The soluble fraction was applied directly to a Ni-NTA column and washed with 50 mM HEPES, 150 mM NaCl. and 30 mM imidazole at pH 7.5. The proteins were eluted by applying a high imidazole elution buffer (50 mM HEPES, 150 mM NaCl and 300 mM imidazole at pH 7.5). Fractions were collected and protein purity was analyzed by sodium dodecyl sulfate polyacrylamide gel electrophoresis (Fig. S3). The protein concentration was determined by the Bradford protein assay measuring the absorbance at 595 nm (Fisher Scientific).

### Generation of a prFMN-containing crude cell lysate

A source of prFMN was generated as previously described (65) with slight modification. The gene for the EcdB prenyl transferase was PCR amplified from the ΔLigAB1:EcDec pK18 plasmid, and cloned into pVP302K. The resulting expression vector was then transformed into B834 *E. coli* containing the pRARE2 plasmid. A single colony of the EcdB B834 *E. coli* was used to inoculate 10 mL of LB media containing kanamycin (50 mg/L) and chloramphenicol (20 mg/L). The culture was incubated for ~18 h at 37°C with shaking (200 rpm). Next, the 10 mL culture was used to inoculate 1 L of TB kanamycin and chloramphenicol media and incubated with shaking for 5 to 6 h until an OD_600_ of 0.7 was reached. The culture was then transferred to 1 L screw top bottle with a magnetic stir bar for anaerobic growth and was amended to include a final concentration of 1% dimethylsulfoxide, 1 mM prenol, 0.1 mM riboflavin, and 0.4 mM IPTG as previously described (65). After incubation overnight (~18 h), the cells were then lysed via sonication and clarified by centrifugation. The resulting crude cell lysate (prFMN lysate) was used as a source of prFMN for the PCA decarboxylase activity assays.

### PCA decarboxylase *in vitro* activity assays

All PCA decarboxylase activity assays were performed in triplicate in the reaction buffer (50 mM HEPES, 150 mM NaCl pH 7.5) using purified enzymes. A stock solution of 25 mM PCA was generated by dissolving 39 mg into 10 mL of reaction buffer. To test the dependence of NadCD activity on prFMN, reactions were initiated by adding 0.1 µM (final concentration) NadCD to a 2 mL (final volume) reaction mixture that contained 1 mM PCA. For reactions that included the prFMN crude cell lysate, the assay mixture also contained 1 mL of prFMN lysate (50% lysate) and 1 mM PCA at a final volume of 2 mL. The enzyme assay was quenched by the addition of 40 µL of 1M HCl to a 0.2 mL aliquot of the reaction mixture at T = 0 and T = 18 h. For temporal analysis of PCA decarboxylase activity by NadCD or EcAroY, those reaction were initiated by the addition of enzyme to final concentration of 0.1 µμM and added to the reaction mixture that included the prFMN crude cell lysate detailed above. Aliquots of 0.2 mL were removed and the reaction terminated as above at various time points. A control reaction of the prFMN lysate reaction mixture without the addition of enzyme was also performed. All reaction products were filtered through 0.22 µm nylon syringe tip filter (Fisher Scientific) and analyzed by LCMS to test for PCA to catechol conversion.

### 1,2 catechol dioxygenase *in vitro* activity assays

Catechol dioxygenase activity was tested in triplicate with either NaCatA or EcCatA in a reaction buffer of 50 mM HEPES, 150 mM NaCl pH 7.5. A stock solution of 0.1 M catechol was freshly prepared by dissolving 22 mg into 2 mL of the reaction buffer. A series of dilutions were performed on the stock solution to obtain a 1 mM catechol working solution. The reactions were performed in a 96-well plate in a total volume of 200 µL with orbital shaking at 28°C using a Tecan infinite M1000 Pro to ensure O_2_ dissolution. The reaction mixture contained 0.5 µM of purified enzyme in reaction buffer and the assay was initiated by the addition of catechol. The formation of *cc*MA was monitored at 260 nm (49) and the resultant data were best fit to a linear equation ([*cc*MA] =*kt* + [*cc*MA]_0_) to yield zeroth order rate constants (*k*) for each enzyme.

### *N. aromaticivorans* extracellular metabolite analysis by HPLC-MS

Extracellular metabolite analysis was carried out on a Shimadzu triple-quadrupole LCMS (Nextera XR HPLC-8045 MS/MS). The mobile phase used a binary gradient with solvent A (0.2% formic acid in water) and solvent B (acetonitrile) using the protocols listed in Tables S7 and S9. The stationary phase used was a Kinetex C18 column (Kinetex 2.6 µm pore size, 100 Å 150 length × 2.1 mm ID, P/N: 00F-4462-AN). Quantification of the metabolites was performed by preparing standard solutions of compounds (Sigma-Aldrich). A series of dilutions were performed to obtain a set of five concentrations for each compound that was within the range of the predicted amount of analyte (Fig. S4 and S7). The compounds were analyzed by both UV/vis absorption and multiple-reaction monitoring. Each of the compounds was quantified using the maximum absorbance wavelength in the UV/visible absorption spectrum and a standard curve was applied using the area under the curve at that maximum absorbance (Tables S8 and S10). The percent yields for *cc*MA were obtained by using the equation below, and the initial aromatic concentrations refer to the aromatic carbon that was used in the growth experiments.


$$percent\;yield=\;\frac{{\left[ccMA\right]}_{final}}{{\left[aromatic\right]}_{initial}}\times 100$$

### Alkaline pre-treatment liquor preparation

The line 15.1 of QsuB poplar was obtained and treated to create APL as previously described (50, 51). The total phenolics in APL were calculated as the sum of the free phenolics and the glycosylated phenolics released after acid treatment of the APL (51). Growth experiments in the presence of APL were performed after adjusting to pH to 7.0 with hydrochloric acid and supplementing with glucose (1 g/L) and ammonium sulfate (1 g/L) (51). The percent yield of *cc*MA production was calculated as indicated above.

## SUPPLEMENTAL MATERIAL

Supplemental information aem.01660-23-s0001.docx Additional experimental details, supplemental figures and tables.

ASM does not own the copyrights to Supplemental Material that may be linked to, or accessed through, an article. The authors have granted ASM a non-exclusive, world-wide license to publish the Supplemental Material files. Please contact the corresponding author directly for reuse.
