# Supplementary material for: Engineering Novosphingobium aromaticivorans to produce cis,cis-muconic acid from biomass aromatics
Source: Appl Environ Microbiol. 2023 Dec 20;90(1):e01660-23. doi: 10.1128/aem.01660-23 (PMC10807440; doi:10.1128/aem.01660-23)
Supplement: Supplemental information — Additional experimental details, supplemental figures and tables. [file aem.01660-23-s0001.docx]

**Supplementary Information**

Engineering *Novosphingobium aromaticivorans* to produce *cc*MA from biomass aromatics

Avery C. Vilbert^ab^, Wayne S. Kontur, Derek Gille, Daniel R. Noguera^abc^ and Timothy J. Donohue^abd^

^a^DOE Great Lakes Bioenergy Research Center, Madison, WI

^b^Wisconsin Energy Institute, University of Wisconsin-Madison, Madison, WI

^c^Department of Civil and Environmental Engineering, University of Wisconsin-Madison, Madison, WI

^d^Department of Bacteriology, University of Wisconsin-Madison, Madison, WI

﻿*Corresponding author: Timothy J. Donohue, [tdonohue@wisc.edu](mailto:tdonohue@wisc.edu)

**Supplemental Tables and Figures:**

**Table S1.** Primers used for protein expression. The regions in bold are complementary to the linearized regions of the pVP302K plasmid. All primers are listed in the 5′ to 3′ orientation.

| **Primers** | **Sequence** | **Plasmid** |
| --- | --- | --- |
| EcdB_pVP_HiFi_F | **GTATTTTCAGAGCGCGATCGCAGGA**ATGAGGCTCATCGTGGGCATGACG | EcdB_pVP302K |
| EcdB_pVP_HiFi_R | **CTAACTTTGTTATTTTCGGCTTTCTG**TCAGATGCCGTCGTTGTTCTCCTGG | EcdB_pVP302K |
| EcAroY_EcdD_pVP_HiFi_F | **GTATTTTCAGAGCGCGATCGCAGGA**ATGCAGAACCCCATCAACGACCTC | EcAroY_D_pVP302K |
| EcAroY_EcdD_pVP_HiFi_R | **CTAACTTTGTTATTTTCGGCTTTCTG**GCTCACAGGAGCGGAGGAATCGTG | EcAroY_D_pVP302K |
| Saro3830_pVP_HIFI_F2 | **GTATTTTCAGAGCGCGATCGCA**ATGCCTGCCACCTTCGCCAG | NaCatA_pVP302K |
| Saro3830_pVP_HIFI_R2 | **CTAACTTTGTTATTTTCGGCTTTCTG**TCAGGCCTGGGCGCGGGTG | NaCatA_pVP302K |
| Saro_3877_8_pVP_F | **gtattttcagagcgcgatcgca**ATGACCATGAACGATCTCCCTAACC | NadCD_pVP302K |
| Saro_3877_8_pVP_R | **CTAACTTTGTTATTTTCGGCTTTCTG**ttattgctggagaatcttgggataac | NadCD_pVP302K |
| EcCatA_pVP_HIFI_F | **GTATTTTCAGAGCGCGATCGCAGGA**ATGTCGAAGAACCCGTCGCAGCA | EcCatA_pVP302K |
| EcCatA_pVP_HIFI_R | **CTAACTTTGTTATTTTCGGCTTTCTG**TTCCTTGACGCGGGCGCGCTTG | EcCatA_pVP302K |

**Table S2.** Primers used for deleting and inserting genes in *N. aromaticivorans*. All Primers are shown in the 5′ to 3′ orientation.

| Genomic modification | PCR reaction | Primers | Notes |
| --- | --- | --- | --- |
| Deletion of Saro_3877-8 | Amplify upstream of Saro_3877 | **Saro_3877-8 Ampl F pk18 HiFi**  CGATTCATTAATGCAGCTGGCACGACAGCGATCTGCTCGCTAAATTGTGGAAGGAG  **Saro_3877-8 HiFi Del R**  GCTGGAGAATCTTGGGATAACCGTTCCCGCCTGTCAGTGTC | Blue and green sequences are complementary to the end regions of linearized pK18mobsacB. Regions in red or purple are complementary to each other. |
|  | Amplify downstream of Saro_3878 | **Saro_3877-8 HiFi Del F**  CTGACAGGCGGGAACGGTTATCCCAAGATTCTCCAGCAATAAGGCTC  **Saro_3877-8 Ampl R pk18 HiFi**  GTTTCTGCGGACTGGCTTTCTAGATGTTCCGTAAAGGTTCCAGTAGCCTAGTCCG |  |
| Deletion of Saro_3873-8 | Amplify upstream of Saro_3873 | **Saro3873-8 pk18 HiFi Ampl-F**  CGATTCATTAATGCAGCTGGCACGACAGGATGTTACGAAGTTCACCTTCACCGGTTC  **Saro3873-8 HiFi Del R**  CTGGAGAATCTTGGGATAACCAGGAGACTTTCCTGCGCGTTTTGG | Blue and green sequences are complementary to the end regions of linearized pK18mobsacB. Regions in red or purple are complementary to each other. |
|  | Amplify downstream of Saro_3878 | **Saro3873-8 HiFi Del F**  CCCAAAACGCGCAGGAAAGTCTCCTGGTTATCCCAAGATTCTCCAGCAATAAGGCTC  **Saro_3877-8 Ampl R pk18 HiFi**  GTTTCTGCGGACTGGCTTTCTAGATGTTCCGTAAAGGTTCCAGTAGCCTAGTCCG |  |
| Inserting Saro_3877-8 into the Saro_2812-3 genomic locus | Linearize pK18msB-ΔSaro2812/3 | **Saro_2812-3 Del R**  CTGACCAGACAGGAGTAGTACCCATG  **Saro_2812-3 Del F**  CAtAGGCCCCTCTCCTTCAGCTTG | Regions in red or purple are complementary to each other. Underlined regions are start or stop codons. The lowercase letter is non-complementary to its initial target sequence. |
|  | Amplify Saro_3877-8 | **Saro3877-8-LigAB R**  CGCATGGGTACTACTCCTGTCTGGTCAGCTTATTGCTGGAGAATCTTGGGATAACGCTG  **Saro3877-8-LigAB F**  CCAAGCTGAAGGAGAGGGGCCTATGACCATGAACGATCTCCCTAACC |  |
| Inserting Saro_3873 and Saro_3877-8 into the Saro_2812-3 genomic locus | Linearize pK18msB-3877-8ΔligAB | **Saro_2812-3 Del F2**  CACAGGCCCCTCTCCTTCAGCTTG  **Saro3877-8_HiFi 3873 F**  GGCAAATGGAAGGGAATTGAGAATGACCATGAACGATCTCCCTAACC | Regions in red or purple are complementary to each other. Underlined regions are start or stop codons. |
|  | Amplify Saro_3873 | **Saro3873 pVP HiFi Ampl F**  CAAGCTGAAGGAGAGGGGCCTGTGAAGCGCATGGTCGTGGGGATTAC  **Saro3873 expr Ampl R**  CATTCTCAATTCCCTTCCATTTGCCCAGCACCCGGAAC |  |
| Deletion of Saro_3857 (*xylE)* | Upstream of *xylE* | **Saro3857_pK18_Amp_F**  gtttctgcggactggctttctacgtgttccgttcattacttcacccagcagggc  **Saro3857_pK18_Hifi_del_R**  ggtctcaaaggctgaacggaaagggcaaggcgatcttctactacgaaaagg | Blue and green sequences are complementary to the end regions of linearized pK18mobsacB. Regions in red or purple are complementary to each other. |
|  | Downstreamof *xylE* | **Saro3857_pK18_Hifi_del_F**  ccttttcgtagtagaagatcgccttgccctttccgttcagcctttgagacc  **Saro3857_pK18_Amp_R**  cgattcattaatgcagctggcacgacagcgaaggtctcatctgatcgaagagcg |  |
| Deletion of saro_3828-3829 | Upstream Fragment | **pK18_amp_delSaro3829_F**  gtttctgcggactggctttctacgtgttctcgccgcaagCTAGGACCGC  **Del_Saro3828-3829_R**  TCGTTGCTTGCCACATCGAAGATCGACggccacaggactaagcgttgc | Blue and green sequences are complementary to the end regions of linearized pK18mobsacB. Regions in red or purple are complementary to each other. |
|  | Downstream  Fragment | **Del_Saro3828-3829_F**  gcaacgcttagtcctgtggccGTCGATCTTCGATGTGGCAAGCAACGA  **pK18_amp_delSaro3829_R**  cgattcattaatgcagctggcacgacagcgttatgtggtgattTCAGCGATCGTCG |  |
| Insertion of catA into Δ*xylE* | Linearizing Δ*xylE* pK18 plasmid | **XylE_catA_ATW_F**  cggactactcccgttatgtggtgatcagcctttgagaccattcctaaagaa  **XylE_catA_ATW_R**  caaggcgatcttctactacgaaaaggcgctgaacgaccgcttcatgacgg | Regions in red or purple are complementary to each other. Bolded region is a ribosomal binding site from the native catA operon. |
|  | Insertion of Saro3830 (*NacatA*) into Δ*xylE* | **XylE_Saro3830_hifi_F**  tttcgtagtagaagatcgccttgcTCAGGCCTGGGCGCGGGTGCGTTCC  **XylE_Saro3830_hifi_R**  tcaccacataacgggagtagtccgATGCCTGCCACCTTCGCCAGTTCCGAT |  |
|  | Insertion of EcCatA into  *xylE* | **XylE_EcCatA_hifi_F** tttcgtagtagaagatcgccttgtcaTTCCTTGACGCGGGCGCGCTTGCCG  **XylE_EcCatA_hifi_R**  **tcaccacataacgggagtagtccg**ATGTCGAAGAACCCGTCGCAGCAGTCG |  |

**Table S3.** Amino acid sequence identity of *N. aromaticivorans* gene products with amino acid similarity to known PCA decarboxylases. Genes from either *Klebsiella pneumoniae* or *Enterobacter cloacae* have been used previously in bacterial engineering for *cc*MA production.

| **Amino Acid Sequence Identity** | **Saro_3873 (NadB)** | **Saro_3877 (NadC)** | **Saro_3878 (NadD)** |
| --- | --- | --- | --- |
| *Enterobacter cloacae* (EcdB) | 47% |  |  |
| *Klebsiella pneumoniae* (KpdB) | 44% |  |  |
| *Enterobacter cloacae* (EcdC) |  | 45% |  |
| *Klebsiella pneumoniae* (KpdC) |  | 46% |  |
| *Enterobacter cloacae* (EcAroY) |  | 31% |  |
| *Klebsiella pneumoniae* (KpAroY) |  | 32% |  |
| *Enterobacter cloacae* (EcdD) |  |  | 25% |
| *Klebsiella pneumoniae* (KpdD) |  |  | 27% |

**Table S4.** Genes used in this study with associated locus tags.

| **Gene** | **Organism** | **Locus tag** |
| --- | --- | --- |
| *ligAB1* | *Novosphingobium aromativicorans* DSM 12444 | Saro_2812-13 |
| *ligAB2* | *Novosphingobium aromativicorans* DSM 12444 | Saro_1233-34 |
| *nadB* | *Novosphingobium aromativicorans* DSM 12444 | Saro_3873 |
| *nadC* | *Novosphingobium aromativicorans* DSM 12444 | Saro_3877 |
| *nadD* | *Novosphingobium aromativicorans* DSM 12444 | Saro_3878 |
| *ecdB* | *﻿Enterobacter cloacae* | ECL_04083 |
| *ecaroY* | *﻿Enterobacter cloacae* | ECL_01944 |
| *ecdD* | *﻿Enterobacter cloacae* | ECL_04081 |
| *nacatA* | *Novosphingobium aromativicorans* DSM 12444 | Saro_3830 |
| *eccatA* | ﻿*Enterobacter cloacae* | HWQ17_22740 |
| *naxylE* | *Novosphingobium aromativicorans* DSM 12444 | Saro_3857 |

**Table S5.** The amino acid sequence identity of CatBCA from *Pseudomonas putida* or *Enterobacter cloacae* compared to homologues encoded by *Novosphingobium aromaticivorans*.

| **Amino Acid Sequence Identity** | **Saro_3828 (NaCatB)** | **Saro_3829 (NaCatC)** | **Saro_3830 (NaCatA)** |
| --- | --- | --- | --- |
| *Enterobacter cloacae* (EcCatB) | 63% |  |  |
| *Pseudomonas putida* (PpCatB) | 62% |  |  |
| *Enterobacter cloacae* (EcCatC) |  | 69% |  |
| *Pseudomonas putida* (PpCatC) |  | 60% |  |
| *Enterobacter cloacae* (EcCatA) |  |  | 43%* |
| *Pseudomonas putida* (PpCatA) |  |  | 49%* |

*only one copy of a *catA* homologue was found in the genome of *N. aromaticivorans*

**Table S6.** DNA sequences of genes inserted into the genome of *N. aromaticivorans*. Start and stop codons are shown in bold. Coding sequences are indicated by upper case and relevant ribosomal binding sites are shown in lower case. DNA sequences in red denote the prenyltransferase coding region, those in green denote the PCA decarboxylase coding region and those in black represent the coding sequence for a gene of unknown function in the EcDec or NaDec decarboxylase gene clusters.

| NaCatA | **ATG**CCTGCCACCTTCGCCAGTTCCGATTCCGTGCAGAAGCTCTTCGATCGCGCCTGCGGTCTTGATTGCGCAGGCGGCAATCCCCGCCTCAAGGCGATCATGCGCGACCTTCTCCAGGCAACGGCCGACATCATCGTCAAGCATGACGTGTCCGAAAGCGAGTTCTGGCAGGCGACCCGCTATCTTGCCGATGGCGCCGGCGAGATCGGCCTGATCGTCCCCGGCATCGGCCTCGAACACTTCCTCGATCTCTACATGGACGCCAAGGACGCCGAAGCCGGCCTCACCGGCGGAACCCCGCGCACGATCGAAGGCCCGCTCTACGTCGCTGGTGCACCGCTGGTGGATGGCAGTGACGAAGTGGACCTGACTTCCGACCCCGACGATACCGACACGCTGCACATGACCGGCACGATCACCGGCCCCGATGGCGAGCCGGTCAAGGACGCGATCCTCCACGTCTGGCACGCGAACAGCAAGGGCTGGTATTCGCACTTCGATCCCACGAGCGAGCAGACCCCGTTCAACAACCGCCGCCGCATCCGCGTCCCCGCCGACGGTCGCTACGCCTTCCGCTCCAAGATGCCGCATGGCTATTCCGTGCCGCCGGGTGGCGCCACCGACGTGCTGATGCAGGCGCTCGGCCGCCACGGCAATCGCCCAGCGCACGTCCACTTCTTCGTCGAGGCGCCGGGCTACCGCACGCTGACCACGCAGATCAACTTCGGCGACGACCCCTTCGCGGCCGACGATTTCGCCTTCGGCACGCGAGAGGGCTTGCTGCCGGTGCCGAGCCGCCAGGGCGATACCGCCCACATCGCGTTCGACTTCCAGCTCCAGCGCGCCCGCTCGGAGGACGAGCAGCGGTTCTCGGAACGCACCCGCGCCCAGGCC**TGA** |
| --- | --- |
| EcCatA | **ATG**TCGAAGAACCCGTCGCAGCAGTCGGAACTCGAAACCCTCCTCGCCATCTCGTCGGGCCTCAACACCGACGGCGGCAACGAACGCGTCAAGCGCGTCATCCACCAGCTCCTCAACGACCTCTGCCACACCATCAAGACCTTCGACATCTCGGACGAAGAATTCTGGATCGCCGTCAACTACCTCAACGAACTCGGCGAACGCAAGGAAGCCGCCCTCCTCGCCGCCGGCCTCGGCCTCGAACACTACCTCGACATGCGCGCCGACGAAAAGGAAGCCGCCTCGGTCTCGCGCGCCGGCACCCCGCGCACCATCGAAGGCCCGCTCTACGTCGCCAACGCCCCGCTCTCGGACCACTTCGCCCGCATGGACGACGGCTCGGAACGCGCCGAAGCCATGTGGCTCCACGGCACCGTCACCGACATCGACGGCAAGCCGGTCGCCGGCGCCATCGTCGACATCTGGCACGCCAACACCCACGGCGGCTACTCGTTCTTCGACCCGTCGCAGTCGGAATACAACCTCCGCCGCCGCGTCAAGACCGCCGCCGACGGCTCGTACGCCGTCCGCTCGATCGTCCCGTGCGGCTACGGCTGCCCGCCGGACGGCCCGACCCAGAAGCTCCTCAACGAACTCGGCCGCCACGGCAACCGCCCGGCCCACATCCACTTCTTCGTCTCGGCCCCGGGCTTCAAGCACCTCACCACCCAGATCAACCTCAACGGCGACCGCTACCTCTGGGACGACTTCGCCTTCGCCACCCGCGAAGAACTCATCGCCGACCCGGTCAAGGTCACCGACTCGACCCTCGCCCGCGAACGCGACATCCACGAACCGCACACCGAAGTCTCGTTCTCGTTCACCCTCGTCAAGGCCGCCGGCGCCGAAGAAGAAGCCCGCGGCAAGCGCGCCCGCGTCAAGGAA**TGA** |
| NaDec | **GTG**AAGCGCATGGTCGTGGGGATTACCGGCGCAACCGGCTCGGTCTATGGTCTTCGCCTGCTTGAGCTGCTGCGCGAGACGGGCGGTTGGGAAACCCATCTGGTAATGTCTCCGGCTGCGCTGCTCAACATTCGCGAGGAACTGCCCGAAGGCAAAGCCCGGCTCGAAGCGCTGGCCGATGTGGTGCACAACGTCCGCAACGTCGGCGCCTCGATCGCCAGCGGTTCGTTCGTATGCGAAGGCATGGCGATTGCGCCCTGTTCGATGCGCACGCTGGGCGCGGTGGCGCACGCCCTGTCCGACAACCTTATCACCCGCGCGGCCGACGTGATGCTGAAGGAACGGCGCCGCCTGGTGATGATCACCCGCGAAGCGCCGCTCAACCTGGCGCACCTGCGCAACATGACGGCCTGCACCGAAATGGGGGCGGTGATCTTCCCCCCGGTGCCGGCCTTCTATGCGCGGCCGACCTCGCTGGCCGACGTGGTCGATCACACCTGCATGCGGGTACTGGATCTGTTCGGGCTTCATGCGAAGTCGGAGAAACGCTGGCAAGGCCTTAGCAAAGAGGCGGCAAGCCTTGTTCCGGGTGCTGGGCAAATGGAAGGGAAT**TGA**ga**ATG**ACCATGAACGATCTCCCTAACCGCGCCCGCTCGATCTCGTCGCTGCGCGACTTCCTCGAACTGCTCGAGGATGCCGGCCAGGCGATCACCTGGAGCGATGCGGTGATGCCCGAACCCGGCGTGCGCAACATAGCCGTCGCCGCATCGCGCGATGCCAACGGCGCGCCGGCGATCGTATTCGACAATATCACCGGTTACCCCGGCAAGCGCTTGGCGGTGGGCGTCCATGGTTCGTGGGACAACATCGCCCTGCTGCTGGGCCGACCTAAAGGCACGACCATCCGCGAGCTTTTCTTCGAGATCGCCGGCCGCTGGGGCGATCAGGAAGCGCAAATCAGCTTTGTCCCAGAAGCCCAGGCCCCGGTGCACGAATGCCGGATCGAACAGGACATCAACCTTTACGATGTCCTGCCGGTCTATCGGATCAACGAATACGATGGCGGGTTCTACATCGGCAAGGCCTCGGTCGCCTCGCGCGATCCGCTCGATCCAGACAATTTCGGCAAGCAGAATGTCGGCATCTATCGCCTGCAGATCCAGGGGCCGGACACCTTCACCCTGATGACGATCCCCTCCCACGACATGGGACGTCAGATCATGGCGGCCGAACGGGAAGGCGTTCCGCTAAAGATTGCGGTCATGCTGGGTAATCATCCCGGCCTTGCGGTGTTTGCTGCCACCCCGATCGGCTACGAGGAATCGGAATATTCCTATGCCTCGGCGATGATGGGCGCGCCAATCCGGCTGACCAAATCGGGCAACGGGATCGACATCCTGGCCGACAGCGAAATCGTGATAGAGGCCGAACTGCAACCGGGTGGACGCGAGCTGGAAGGGCCGTTCGGCGAATTCCCCGGTTCCTACAGCGGCGTGCGCAAGGCGCCGATCTTCAAGGTCACGGCGGTGTCGCACCGGCGCGATCCGATCTTCGAGAACATTTACATCGGGCGCGGCTGGACCGAGCACGATACGCTGATCGGCCTGCACACCTCCGCCCCGATCTATGCCCAGCTGCGCCAGAGCTTCCCCGAAGTCACCGCGGTCAACGCGCTTTACCAGCACGGACTGACCGGGATCATCTCGGTCAAAAACCGCATGGCCGGCTTTGCCAAGACGGTCGCGCTGCGCGCGCTGAGCACGCCGCACGGCGTGATGTACCTCAAGAACCTGATTATGGTCGATGCCGATGTCGATCCGTTCGATCTCAACCAAGTGATGTGGGCGCTTTCGACCCGCACCCGTGCGGACGATATCATCGTGCTGCCCAACATGCCTGCCGTGCCGATCGATCCTTCGGCAGTGGTCCCGGGCAAGGGGCACCGCCTGATCATCGACGCGACCAGCTATCTCCCGCCCGATCCGGTGGGTGAAGCGCACCTTGTCACCCCGCCGACCGGGGACGAGATCGACGCCCTGAGCAAGCGGATCCGCGAAATGCAGCTGGGAGCCCTGTC**ATGA**CCACCACCGTCTGCGGGCGCTGCAAATCGAGCGGCGCTGTCACCGATCATCAGGGCAGGCAGGACGGCGCGGTCGTGTGGACGATCCTGCGCTGCCCGACCTGCAACTTTTCCTGGCGCGACAGCGAACCGGCCCGCGCTATCGACCCGGCTGTGCGCTCGGCCGATTTCGCCGTCGATGTCGGCGATCTCCAGCGTTATCCCAAGATTCTCCAGCAA**TAA** |
| EcDec | **ATG**AGGCTCATCGTGGGCATGACGGGAGCCACGGGCGCTCCGCTTGGCGTGGCCCTCCTGCAGGCGCTCCGCGACATGCCCGAGGTTGAAACCCATCTGGTGATGTCGAAGTGGGCGAAGACCACCATCGAGCTGGAAACGCCGTATACCGCGCAGGACGTCGCTGCCCTGGCCGACGTCGTCCACAGCCCTGCCGATCAGGCAGCCACCATCTCGTCGGGCTCGTTCCGCACCGATGGCATGATCGTCATTCCCTGCAGCATGAAGACGCTTGCAGGCATTCGCGCGGGCTATGCCGAAGGGCTTGTCGGTCGTGCGGCAGATGTTGTGCTGAAAGAAGGTCGCAAGCTGGTGCTGGTCCCGCGCGAAACGCCGCTCAGCACCATCCATCTGGAGAACATGCTCGCGCTTTCCCGCATGGGGGTGGCGATGGTGCCGCCCATGCCCGCGTACTACAACCATCCGCAAACCGCCGACGACATCACCCAGCACATCGTGACCCGCGTCCTCGACCAGTTCGGTCTGGAGCACAAGAAGGCACGTCGCTGGAATGGCCTGCAGGCGGCGAAGCACTTCAGCCAGGAGAACAACGACGGCATC**TGA**tgctgggcaaatggaagggaattgaga**ATG**CAGAACCCCATCAACGACCTCCGCTCTGCCATCGCGCTGCTGCAACGCCATCCCGGTCACTATATCGAAACCGACCACCCGGTCGATCCCAATGCTGAACTGGCGGGCGTCTATCGCCATATCGGCGCGGGCGGTACCGTCAAACGCCCCACCCGCACGGGCCCGGCCATGATGTTCAACAGCGTGAAGGGCTACCCTGGCTCCCGCATCCTGGTCGGTATGCATGCCAGCCGGGAACGCGCGGCGCTTCTGCTGGGCTGTGTCCCCTCGAAGCTGGCACAGCACGTCGGTCAGGCGGTGAAGAACCCGGTTGCACCGGTGGTGGTTCCAGCCTCGCAGGCACCGTGCCAGGAGCAGGTCTTCTATGCCGACGATCCGGACTTCGACCTGCGTAAGCTGCTTCCGGCCCCGACCAACACGCCGATTGATGCAGGCCCGTTCTTCTGCCTGGGGCTGGTCCTGGCAAGCGATCCGGAAGACACCTCGCTGACCGATGTGACCATTCACCGTCTCTGCGTGCAGGAGCGAGACGAACTCTCGATGTTCCTTGCCGCCGGCCGCCATATCGAAGTCTTTCGCAAGAAGGCCGAAGCGGCGGGCAAACCGCTGCCGGTCACCATCAACATGGGACTTGACCCGGCTATCTACATAGGGGCCTGCTTCGAAGCGCCCACCACGCCCTTCGGTTACAACGAGCTTGGCGTTGCCGGGGCACTCCGCCAGCAACCGGTGGAGCTGGTCCAGGGCGTAGCGGTCAAGGAGAAAGCGATCGCGCGGGCGGAAATCATCATCGAGGGCGAACTGCTTCCCGGCGTGCGCGTCCGCGAAGATCAGCACACCAACACCGGCCACGCCATGCCGGAGTTCCCGGGCTACTGCGGCGAGGCGAATCCGTCGCTGCCGGTGATCAAGGTGAAAGCCGTGACGATGCGAAACCATGCGATCCTGCAGACGCTGGTGGGCCCTGGCGAAGAGCACACCACGCTTGCCGGTCTGCCGACCGAGGCCAGCATTCGCAACGCGGTCGAAGAGGCCATTCCCGGCTTTCTGCAGAACGTCTACGCCCACACCGCCGGAGGCGGTAAGTTCCTCGGCATCCTACAGGTGAAGAAGCGCCAGCCGTCGGACGAAGGACGTCAGGGCCAGGCGGCACTCATCGCCCTGGCCACCTATTCCGAGCTGAAGAACATCATCCTCGTGGACGAAGACGTGGACATCTTCGACAGCGACGACATCCTGTGGGCAATGACCACCCGCATGCAGGGCGATGTGAGCATCACCACGCTTCCGGGGATCCGCGGCCACCAGCTGGATCCGTCGCAGTCGCCGGACTACAGCACCTCGATCCGTGGAAACGGCATCTCCTGCAAGACTATCTTCGACTGCACGGTGCCGTGGGCGCTGAAGGCGCGGTTCGAACGGGCGCCGTTCATGGAGGTCGACCCCACACCGTGGGCGCCGGAGCTGTTCAGCGACAAGAAG**TGA**cagctgggagccctgtc**ATG**ATCTGCCCGCGCTGCGCCGACGAGCAGATCGAGGTCATGGCCACCAGCCCGGTGAAGGGCATCTGGACCGTCTACCAGTGCCAGCACTGCCTGTACACCTGGCGGGACACCGAACCGCTTCGTCGCACCTCGCGCGAGCACTATCCCGAAGCGTTCCGCATGACGCAGAAGGACATCGATGAAGCGCCGCAGGTGCCCACGATTCCTCCGCTCCTG**TGA**gctgaccagacaggagtagtaccc |

**Figure S1.** Catechol to *cc*MA production by NaCatA. The reaction was initiated with 0.5 μM enzyme with either 25 μM or 50 μM catechol in 50 mM HEPES 150 mM NaCl pH 7.5. The production of *cc*MA was measured by the increase in absorbance at 260 nm.

**Figure S2**. Relative transcript abundance of *catA* (Saro_3830) and *xylE* (Saro_3857) when *N. aromaticivorans* strain 12444 is grown with either glucose (blue), protocatechuic acid (red) or vanillic acid (green) as a carbon source.

**Figure S3.** SDS-PAGE gel of purified recombinant EcAroY/D (a), NadCD (b), EcCatA (c) and NaCatA (d). The box highlights the purified protein.

| Time (min) | %B conc |
| --- | --- |
| 0.05 | 0 |
| 1.9 | 0 |
| 3 | 0 |
| 3.25 | 2 |
| 3.5 | 4 |
| 3.75 | 6 |
| 4 | 8 |
| 4.25 | 10 |
| 4.5 | 12 |
| 4.75 | 14 |
| 5 | 16 |
| 6 | 20 |
| 7.5 | 50 |
| 8 | 95 |
| 9 | 95 |
| 9.25 | 5 |
| 10 | 0 |
| 11 | End of Run |

**Table S7.** Liquid chromatography (LC) program for elution of phenolics using method 1.

**Figure S4.** Chromatogram of the indicated aromatics used in method 1 (see Materials and Methods) to quantify the aromatics at 263 nm.

| **Compound** | **Retention Time** | **Maximum UV/vis absorption** | **MRM m/z** |
| --- | --- | --- | --- |
| Vanillic acid | 6.692 | 263 nm | 167.00>152.10 |
| PCA | 4.473 | 263 nm | 153.00>109.10 |
| Catechol | 4.701 | 275 nm | 109.20>91.10 |
| ccMA 1 | 5.570 | 263 nm | 141.15>97.10 |
| ccMA 2 | 6.217 | 263 nm | 141.15>97.10 |

**Table S8.** Multiple Reaction Monitoring (MRM) transitions and maximum UV/vis absorption data used to quantify phenolics in method 1 (see Materials and Methods).

**Figure S5.** Representative HPLC traces of extracellular aromatics using LC method 1 from *N. aromaticivorans* strains 12444 (a), ΔligAB1/2 (b) or 12444_PCA (c) grown with 2 mM vanillic acid and 10 mM glucose as carbon sources. The black trace represents the sample taken at T = 0 and the red trace represents the sample taken at T= 50 h. All identified aromatics are labeled.

**Figure S6.** Representative HPLC traces of extracellular aromatics using LC method 1 from *N. aromaticivorans* strains LigAB1_EcDec (a) or LigAB1_NaDec (b) grown with 2 mM vanillic acid and 10 mM glucose as carbon sources. The black trace represents the sample taken at T = 0 and the red trace represents the sample taken at T= 48 h. All identified aromatics are labeled.

| Time (min) | %B conc |
| --- | --- |
| 0.05 | 5 |
| 3.5 | 8 |
| 4 | 11 |
| 5.5 | 12 |
| 6.5 | 14 |
| 7 | 30 |
| 7.5 | 50 |
| 8 | 75 |
| 8.5 | 95 |
| 9.25 | 95 |
| 9.5 | 5 |
| 10 | 5 |
| 10.5 | 1 |
| 11 | End of run |

**Table S9.** LC program for elution of phenolics in method 2 (see Materials and Methods).

**Figure S7.** Chromatogram of the commercial compounds used as aromatics to quantify the aromatics present in Qsub poplar APL measured at 263 nm with chromatographic method 2. The aromatics measured with this method (1 mM final concentration) are shown and labeled above each peak with protocatechuic acid (PCA), *cis,*cis-muconic acid (*cc*MA), 4-hydroxybenzoic acid (4-HBA), vanillic acid, vanillin and 4-coumaric acid (4-CA) and ferulic acid,

| **Compound** | **Retention Time** | **Maximum UV/vis absorption** | **MRM m/z** |
| --- | --- | --- | --- |
| 4-CA | 7.186 | 309 nm | 163.00>119.10 |
| 4-HBA | 3.732 | 254 nm | 136.90>93.10 |
| Catechol | 3.049 | 275 nm | 109.20>91.10 |
| ccMA 1 | 2.400 | 263 nm | 141.15>97.10 |
| ccMA 2 | 3.367 | 263 nm | 141.15>97.10 |
| Ferulic Acid | 8.283 | 322 nm | 193.00>134.10 |
| PCA | 2.277 | 263 nm | 153.00>109.10 |
| Vanillic acid | 5.473 | 263 nm | 167.00>152.10 |
| Vanillin | 6.647 | 309 nm | 151.00>136.00 |

**Table S10.** Multiple Reaction Monitoring (MRM) transitions and maximum UV/vis absorption data used to quantify extracellular aromatics in Qsub poplar APL method 2.

**Figure S8.** Representative HPLC traces of extracellular aromatics using LC method 2 from *N. aromaticivorans* strains of EcDec_*cc*MA (a) or NaDec_*cc*MA (b) grown with 2 mM protocatechuic acid (PCA) and 10 mM glucose. The black trace represents the sample taken at T = 0 and the blue trace represents the sample taken at T= 72 h. All identified aromatics are labeled.

**Figure S9.** Aromatics identified in Qsub Poplar APL before and after acid hydrolysis. The HPLC trace (panel a) is shown with the identified aromatics protocatechuic acid (PCA), vanillic acid, and 4-hydroxybenzoic acid (4-HBA). The corresponding calculated concentrations of aromatics identified before (red bar) and after (blue bar) acid hydrolysis are shown in panel b.

| **Aromatic** | **Before acid hydrolysis** | **After acid hydrolysis** |
| --- | --- | --- |
| PCA | 0.00 $\pm$ 0.000 mM | 0.31 $\pm$ 0.025 mM |
| Vanillic Acid | 0.04 $\pm$ 0.006 mM | 0.10 $\pm$ 0.028 mM |
| 4-HBA | 0.04 $\pm$ 0.001 mM | 0.03 $\pm$ 0.014 mM |
| Vanillin | ND | ND |
| 4-CA | Trace | ND |
| Ferulic Acid | Trace | ND |

**Table S11.** Concentration of aromatics in Qsub Poplar APL before and after acid hydrolysis. ND represents that the phenolic was not detected and trace indicates that a compound that co-migrated with a commercial standard was detected but at too low of levels to be quantified.

**Figure S10.** Representative HPLC traces of extracellular aromatics using LC method 2 from *N. aromaticivorans* strains EcDec_*cc*MA (a) or NaDec_*cc*MA (b) grown with Qsub APL popular as a carbon source. The black trace represents the sample taken at T = 0 and the red trace represents the sample taken at T= 48 h. All identified aromatics are labeled.

**Supplemental Methods:**

**Construction of plasmids for generating deletions of Saro_3877-8 and Saro_3873-8.** Regions of *N. aromaticivorans* genomic DNA containing ~1000 base pairs (bp) upstream and downstream of the genes to be deleted were amplified via PCR using the primers listed in Table S2. Plasmid pK18mobsacB was linearized via PCR as previously described.(1) The upstream and downstream flanking regions for each gene were combined with linearized pK18mobsacB using the NEBuilder HiFi Assembly system (New England Biolabs, Ipswich, MA) to produce a plasmid in which the upstream and downstream DNA sequences are adjacent, with no intervening coding region. For the strain in which Saro_3873-8 was deleted, the deleted region begins two bp before the start codon of Saro_3873. For the strain in which Saro_3877-8 was deleted, the deleted region begins one bp before the start codon of Saro_3877. In both strains, the deletion extends to the same point near the end of Saro_3878: the final 26 bp of Saro_3878 remain in the *N. aromaticivorans* genome.

**Construction of a plasmid to insert Saro_3877-8 into the Saro_2812-3 locus of the *N. aromaticivorans* genome.** Plasmid pK18mobsacB/ΔSaro2812/3 (2) which contains ~1,000 bp genomic regions upstream and downstream of Saro_2812-3, was linearized via PCR using primers shown in Table S2. Concurrently, Saro_3877-8 was amplified from *N. aromaticivorans* genomic DNA via PCR using primers containing upstream regions complementary to linearized pK18mobsacB/ΔSaro2812/3 (Table S2). The linearized pK18mobsacB/ΔSaro2812/3 and the Saro_3877-8 fragment were combined using the NEBuilder HiFi Assembly system (New England Biolabs, Ipswich, MA) to produce plasmid pK18mobsacB-3877-8ΔligAB, in which Saro_3877-8 was placed between the DNA regions that naturally flank Saro_2812-3. The Saro_3877 start codon is in the natural position for the Saro_2813 start codon, and the Saro_3878 stop codon is in the natural position of the Saro_2812 stop codon, with an additional C following the Saro_3878 stop codon that does not naturally follow the Saro_2812 stop codon. The Saro_2814 stop codon (UGA), which naturally overlaps with the Saro_2813 start codon, remains intact and now overlaps with the Saro_3877 start codon.

**Construction of a plasmid to insert Saro_3873 and Saro_3877-8 into the Saro_2812-3 locus of the *N. aromaticivorans* genome.** Plasmid pK18mobsacB-3877-8ΔligAB was linearized via PCR using primers in Table S2. Concurrently, Saro_3873 was amplified from *N. aromaticivorans* genomic DNA via PCR using primers containing upstream regions complementary to linearized pK18mobsacB-3877-8ΔligAB (Table S2). The linearized pK18mobsacB-3877-8ΔligAB and the Saro_3873 fragments were combined using the NEBuilder HiFi Assembly system (New England Biolabs, Ipswich, MA) to produce plasmid pK18mobsacB-3873/7-8ΔligAB, in which Saro_3873 and Saro_3877-8 formed an artificial operon between the DNA regions that naturally flank Saro_2812-3 (Table S6). The Saro_3873 start codon is in the natural position for the Saro_2813 start codon. The 2 bp intergenic region normally between Saro_3873 and Saro_3874 follows the Saro_3873 stop codon, and is now followed by the Saro_3877 start codon.

**Plasmids for inserting genes encoding the EcdB, EcAroY, and EcdD proteins from *Enterobacter cloacae* into the Saro_2812-3 locus of the *N. aromaticivorans* genome.** DNA fragments containing the genes coding for EcdB (NCBI Accession: ADF63617), EcAroY (NCBI Accession: ADF61496), and EcdD (NCBI Accession: ADF63615) from *Enterobacter cloacae* (Table S4 and S6) were ordered as gBlocks from Integrated DNA Technologies (Coralville, IA). Genes were constructed to have codon usage frequencies similar to those of other genes in *N. aromaticivorans* (calculated from several genes in the genome), but without making the GC content of the fragments too high for the gBlock synthesis process. The DNA sequence of the operon was split between two gBlocks (EcdB-aroY-D-NaligAB_up and EcdB-aroY-D-NaligAB_down); each gBlock contained a sequence at one end that matches one of the ends of linearized pK18mobsacB-ΔSaro2812/3; the other ends of the gBlocks match each other. The two gBlocks were combined with linearized pK18mobsacB-ΔSaro2812/3 using the NEBuilder HiFi Assembly system (Table S6). The *ecdB* start codon is in the natural position for the Saro_2813 start codon, and the *ecdD* stop codon is in the natural position of the Saro_2812 stop codon.

**Construction of plasmids Δ*xylE*_pK18mobsacB and ΔcatBC_pK18mobsacB.** Primers for deletion of *catBC* and *xylE* (Table S2) were designed to amplify two ~1000 bp regions both downstream and upstream of the desired gene deletion in the genome of *N.aromaticivorans*. For each plasmid, these two amplified regions were combined with linearized pK18mobsacB-MCS1 as described above using the NEBuilder HiFi Assembly system. For Δ*catBC*, the deletion begins 1 bp upstream of the start codon of *saro_3828* with the final 133 bp of *saro_3829* remaining in the genome. The deleted region of Δ*xylE* begins 9 bps upstream of the start codon of *saro_3857* (*xylE*) with the final 62 bp of *saro_3857* remaining in the genome.

**Construction of plasmids Δ*xylE_eccatA*­ and Δ*xylE_nacatA*­ pK18mobsacB.** For insertion of *catA* into the *xylE* locus of *N. aromaticivorans,* the **Δ**xylE_pK18mobsacB plasmid was first linearized using XylE_catA_ATW_F and XylE_catA_ATW_R. The XylE_CatA_ATW_F primer contained a ~ 20 bp region that is located upstream of the native *nacatA* consistent with the ribosomonal binding site (RBS) for *nacatA.* Primers listed in SI Table 2 were used to amplify either *eccatA* or *nacatA* out of the pVP302K vector with overhangs corresponding to the linearized ΔxylE_pK18mobsacB. Next, either fragment *eccatA* or *nacatA* was combined with the linearized ΔxylE_pK18mobsacB using the NEBuilder HiFi Assembly system.

**Conjugation Procedure:** Bacterial conjugations using either *E. coli* S17 or WM6026 donor cells with *N. aromaticivorans* recipients were performed essentially as previously described(3, 4) with slight modifications. Cultures of *E. coli* WM6026 cells were grown in LB + kanamycin (kan) and 0.3 mM diaminopimelic acid (DAP) while *N. aromaticivorans* strains were grown in 10 mM glucose SMB media. Both strains were grown overnight at 30 °C in 5 mL of media in an 18 x 150 mm culture tube. The overnight cultures were then diluted 1:1 and grown to mid-log phase. The cell density of the donor and recipient cultures was then measured and the cultures were diluted such that the cell density of both the recipient and the donor were equal. Next, 2 mL of the donor and 1 mL of the recipient culture was subsequently washed and pelleted prior to mixing in a 2:1 donor to recipient cell ratio. The mixed cells were then suspended into 0.1 mL of LB DAP and incubated at 30 °C for 4 h. After incubation, the cells were then pelleted by centrifugation and resuspended into 0.5 mL SMB supplemented with 10mM glucose before incubating and shaking for an additional hour at 30 °C. The cells were plated onto SMB Kan and transconjugant colonies formed within 3 to 4 days. Double crossover variants were selected as previously described(3) from SMB 10% sucrose plates. PCR amplified regions of the targeted genes were sequenced to confirm the mutation.

**References:**

1. Kontur WS, Bingman CA, Olmsted CN, Wassarman DR, Ulbrich A, Gall DL, Smith RW, Yusko LM, Fox BG, Noguera DR, Coon JJ, Donohue TJ. 2018. Novosphingobium aromaticivorans uses a Nu-class glutathione S-transferase as a glutathione lyase in breaking the -aryl ether bond of lignin. J Biol Chem 293:4955–4968.

2. Perez JM, Kontur WS, Gehl C, Gille DM, Ma Y, Niles A V., Umana G, Donohue TJ, Noguera DR. 2021. Redundancy in Aromatic O-Demethylation and Ring-Opening Reactions in Novosphingobium aromaticivorans and Their Impact in the Metabolism of Plant-Derived Phenolics. Appl Environ Microbiol 87:1–23.

3. Perez JM, Kontur WS, Alherech M, Coplien J, Karlen SD, Stahl SS, Donohue TJ, Noguera DR. 2019. Funneling aromatic products of chemically depolymerized lignin into 2-pyrone-4-6-dicarboxylic acid with: Novosphingobium aromaticivorans. Green Chem 21:1340–1350.

4. Cecil JH, Garcia DC, Giannone RJ, Michener JK. 2018. Rapid, Parallel Identification of Catabolism Pathways of Lignin-Derived Aromatic Compounds in Novosphingobium aromaticivorans. 84:1-13. https://doi.org/10.1128/AEM.
